# Zn(II) and Cu(II) Coordination Enhances the Antimicrobial Activity of Piscidin 3, but Not That of Piscidins 1 and 2

**DOI:** 10.1021/acs.inorgchem.4c01659

**Published:** 2024-07-01

**Authors:** Miller Adriana, Mikołajczyk Aleksandra, Bellotti Denise, Garstka Kinga, Wątły Joanna, Hecel Aleksandra, Wieczorek Robert, Matera-Witkiewicz Agnieszka, Rowińska-Żyrek Magdalena

**Affiliations:** † Faculty of Chemistry, University of Wroclaw, ul. F. Joliot-Curie 14, 50-383 Wroclaw, Poland; ‡ Screening of Biological Activity Assays and Collection of Biological Material Laboratory, Wroclaw Medical University Biobank, Faculty of Pharmacy, Wroclaw Medical University, ul. Borowska 211a, 50-556 Wroclaw, Poland; § Department of Chemical, Pharmaceutical and Agricultural Sciences, University of Ferrara, Via Luigi Borsari 46, 44121 Ferrara, Italy

## Abstract

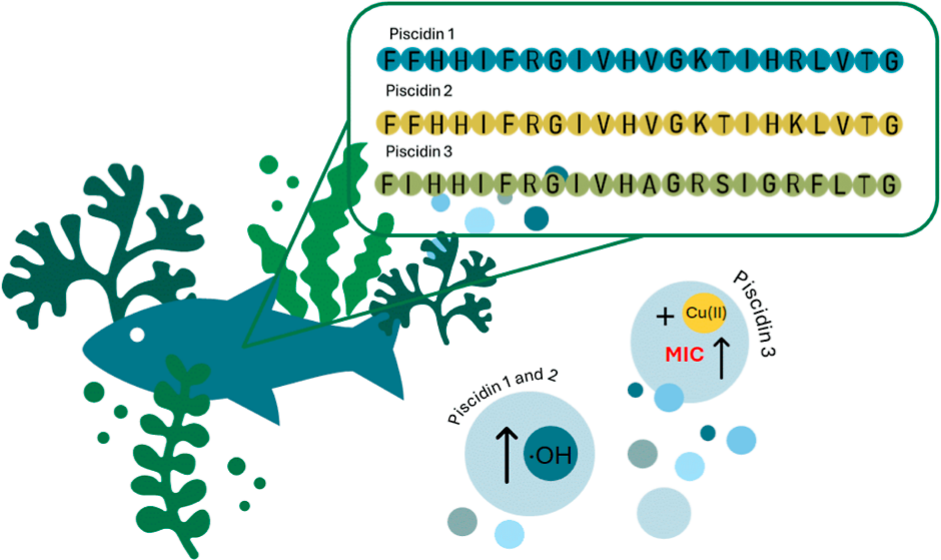

Piscidins, antimicrobial peptides isolated from fish, are potent against a variety of human pathogens; they show minimum inhibitory concentration values comparable to those of commercially used antimicrobials. Piscidins 1 and 2 are generally more effective than piscidin 3 when applied alone; the contrary is observed for their metal complexes: Zn(II) and Cu(II) coordination does not enhance the efficacy of piscidins 1 and 2, while a moderate enhancement is observed for piscidin 3. All three piscidins bind Cu(II) in a so-called albumin-like binding mode, while for Zn(II) complexes, two coordination modes are observed: piscidins 1 and 2 bind Zn(II) by imidazole nitrogens from His4, His11, and His17 side chains; piscidin 3 coordinates Zn(II) by His3, His4, and His11 imidazole nitrogens and additionally supports the interaction, formed by carbonyl oxygen from His4. Most likely, the high antimicrobial activity of piscidin complexes is due to neither the stability of their complexes nor the change in their secondary structure. Copper(II) complexes with piscidins 1 and 2 can form hydroxyl radicals, which could be responsible for the antimicrobial membrane damaging activity of these complexes. Clearly, a different mechanism (most likely an intercellular targeted one) is observed for piscidin 3 metal complexes; in most cases, the coordination of Cu(II) and Zn(II) enhances the antimicrobial potency of piscidin 3, showing that not only piscidin 3 alone but also its metal complexes have a different mode of action than piscidins 1 and 2.

## Abstract 2

Piscidins, antimicrobial peptides isolated from fish, show MIC values against a variety of human pathogens comparable to those of commercially used antimicrobials. Most likely, the high antimicrobial activity of piscidin complexes is due neither to their stability nor to the change in their secondary structure. Copper(II) complexes with piscidins 1 and 2 can form membrane-damaging hydroxyl radicals. Clearly, a different mechanism (most likely an intercellular targeted one) is observed for piscidin 3 metal complexes.

## Introduction

Increasing antimicrobial resistance has been an emerging problem for global health in the last years. According to WHO, a growing number of infections were noticed as significantly harder or even impossible to treat with currently used antimicrobials.^1,2^ Microbes become resistant to antibiotics by developing their defensive strategies, such as drug target modification, drug uptake inhibition, drug removal by active efflux, and drug enzymatic inactivation.^3^ Among the main reasons for growing resistance is misuse or overuse of antimicrobials and the strategy to cope with this problem is based on preventing infections, optimizing the usage of antimicrobial drugs and searching for new therapeutics.^1,2^

The development of new antimicrobial agents has slowed in the last years. Since first July 2017, FDA (U.S. Food and Drug Administration) together with EMA (European Medicines Agency) have approved 11 new antibiotics, but 80% of them come from existing classes with well-known resistance mechanisms, which can suggest a quick appearance of resistance against these new drugs. In 2020, 43 antibacterials were in clinical trials, and 26 of them focused on pathogens assigned by WHO with the highest priority.^4^ Additionally, in preclinical studies there were 292 antibacterial agents, 29 of which belong to the group called as antimicrobial peptides (AMPs)^4^—a widespread and diverse group of compounds naturally present in many multicellular organisms, where they take part in reactions of the innate immune system.^5,6^ Currently, around 3940 AMPs are deposited in specific databases.^7^ They can be classified by their biological source (microbes, plants, animals, or fungi), presence of specific amino acids (and the resulting properties such as hydrophobicity or charge), secondary structure (e.g., α-helical or beta-sheet), activity (antibacterial, antiviral, antifungal, antitumor, etc.), or molecular targets (cell surface or intracellular targets).^8,9^ Most of them are α-helical, cationic peptides consisting of 10–100 amino acids. Single peptides often can show multiple modes of action, which is one of the probable reasons for the minimal antimicrobial resistance toward AMPs despite millions of years of coexistence with pathogens.^6^

One of the strategies that marine organisms have developed in order to protect themselves from bacteria and fungi is based on the production of AMPs, which play a key role in defense against surrounding pathogens, making AMPs particularly important for simple organisms, which did not develop an adaptive immune system.^6,10^ Moreover, AMPs from aquatic organisms were naturally designed to maintain their functionality in extremely demanding conditions, such as high salinity and a wide range of temperature and pressure.^10−12^ Resistance to high salinity is an especially valuable feature, as it suggests the maintenance of biological functions in highly saline body fluids such as saliva, gastrointestinal fluid, or serum.^10^ For this purpose, the stability of numerous AMPs is enhanced by the presence of specific post-translational modifications, such as bromination, chlorination, C-terminal amidation, hydroxylation, or disulfide bonds.^10^ The majority of these alterations increases the proteolysis peptides resistance, which, together with the endurance toward high salinity, makes them attractive potential candidates for new drugs.^10,13^

**Figure 1:**
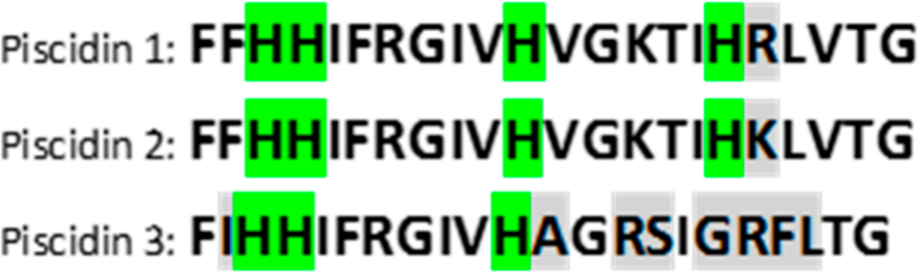
Amino acid sequence of piscidins 1, 2 and 3,^7,16^ His are highlighted in green, and differences in the sequences are highlighted in gray.

In this work, we focus on three AMPs from the piscidin family: piscidins 1, 2, and 3 (Figure 1), which recently have gained a decent amount of scientific attention.^14,15^

Piscidins were originally isolated from the mast cells of hybrid striped bass (Morone saxatilis x Morone chrysops).^16^ They are α-helical, histidine-rich peptides and can exist in a C-terminally amidated and nonamidated form.^17^ So far, the activity of these compounds has been reported not only against fish parasites but also against human pathogens (bacteria, fungi, and viruses) or even cancer cells.^14,18−21^ Despite their high sequence similarity, piscidins show different modes of action: while piscidin 1 most likely presents a membrane-disrupting mechanism, piscidin 3 is thought to prefer intracellular targets, such as DNA.^17^ The reason for these differences is most likely the His17 residue, present only in piscidins 1 and 2. Piscidin 3, lacking this residue, binds to the membrane surface and directs its N-terminus toward the bilayer interior, penetrating the membrane in a more shallow manner than piscidin 1, which penetrates the entire lipid bilayer, leading to the desegregation of the membrane regions, drawing water into the bilayer core, thinning and damaging the membrane. Piscidin 3 passes through membranes most likely by forming defects too minor or short-lived to induce significant harm and leakage.^22^

Interestingly, in some cases, metal–peptide interactions can play a key role in the immune response. For example, peptides can capture all available metal ions, thus preventing pathogens from obtaining these metal nutrients, necessary for their survival and virulence (this process is referred to as “nutritional immunity”).^23^ On the other hand, metal ions can work as boosters for peptides’ antimicrobial activity. This enhancement can be caused, for example, by the change of the peptides’ charge or secondary structure.^9,23^

For this reason, we studied Zn(II) and Cu(II) complexes of piscidins 1, 2 and 3, obtaining information about their stoichiometry, geometry, thermodynamic properties, and the influence of metal binding on the biological activity of piscidins.

## Experimental Methods

### Materials

All peptides (NH_2_–FFHHIFRGIVHVGKTIHRLVTG-COOH, NH_2_–FFHHIFRGIVHVGKTIHKLVTG-COOH, and NH_2_–FIHHIFRGIVHAG-RSIGRFLTG-COOH) were purchased from KareBay Biochem (USA) (certified purity: 98%) and were used as received.

The carbonate-free stock solutions of 0.1 M NaOH were purchased from Sigma-Aldrich and then potentiometrically standardized with the primary standard potassium hydrogen phthalate (99.9% purity).

Cu(II) and Zn(II) perchlorate hexahydrates were extra pure products (Sigma-Aldrich). The concentrations of their stock solutions were determined by inductively coupled plasma mass spectrometry. The carbonate-free stock solution of 0.1 M NaOH (Merck) was potentiometrically standardized with potassium hydrogen phthalate (Sigma-Aldrich). All of the samples were prepared with freshly doubly distilled water. The ionic strength (*I*) was 40 mM SDS (Sigma-Aldrich).

### Mass Spectrometry

High-resolution mass spectra were recorded on a Bruker Apex Ultra FT-ICR (Bruker Daltonik, Bremen, Germany), equipped with an Apollo II electrospray ionization source with an ion funnel and an LCMS-9030 qTOF Shimadzu (Shimadzu, Kyoto, Japan) device, equipped with a standard ESI source and the Nexera X2 system. Both mass spectrometers were operated in the positive ion mode. The instrumental parameters for Bruker Apex Ultra FT-ICR were as follows: scan range *m*/*z* 100–2000, dry gas–nitrogen, temperature 473 K, and ion energy 5 eV. The capillary voltage was optimized to the highest S/N ratio and it was 4200 V. For LCMS-9030 qTOF Shimadzu, the instrumental parameters were as follows: scan range *m*/*z* 100–2000, nebulizing gas nitro-gen, nebulizing gas flow 3.0 L/min, drying gas flow 10 L/min, heating gas flow 10 L/min, interface temperature 300 °C, desolvation line temperature 400 °C, detector voltage 2.02 kV, interface voltage 4.0 kV, collision gas argon, mobile phase (A) H_2_O + 0.1% HCOOH, (B) MeCN + 0.1% HCOOH, and mobile phase total flow 0.3 mL/min. The injection volume was optimized depending on the intensity of the signals observed on the mass spectrum within the range of 0.1 to 3 μL. The samples were prepared in a 1:1 methanol–water mixture with a M^2+^:L molar ratio 1:1, [ligand] = 3 × 10^–4^ M, pH 6. The samples were infused at a flow rate of 3 μL min^–1^. The instrument was calibrated externally with a Tunemix mixture (Bruker Daltonik, Germany) in quadratic regression mode. Data were processed using Bruker Compass DataAnalysis 4.0 and ACDLabs Spectrus Processor v2021.1.3 programs. The mass accuracy for the calibration was better than 5 ppm, enabling together with the true isotopic pattern (using SigmaFit) an unambiguous confirmation of the elemental composition of the obtained complex.

### Potentiometry

Deprotonation constants for ligand and stability constants for Zn(II), and Cu(II) complexes of the peptides were calculated from pH-metric titration curves obtained over the pH range 2–11 at *T* = 298 K in water solution of 4 mM HClO_4_ and ionic strength 40 mM (SDS) using a total volume of 3 mL. The potentiometric titrations were performed using a Metrohm Titrando 905 titrator and a Mettler Toledo InLab Micro combined pH electrode. The thermostabilized glass-cell was equipped with a magnetic stirring system, a microburet delivery tube, and an inlet–outlet tube for argon. Solutions were titrated with 0.1 M carbonate-free NaOH. The electrodes were calibrated daily for hydrogen ion concentration by titrating HClO_4_ with NaOH under the same experimental conditions as above. The purities and exact concentrations of the peptide solutions were determined by the Gran method.^24^ The peptide concentration was 0.5 mM and the Zn(II)- and Cu(II)-to-peptide ratio was 1:1.

The standard potential and the slope of the electrode couple were computed by means of the GLEE program.^25^ The HYPERQUAD 2006^26^ program was used for the stability constant calculations. The standard deviations were computed by HYPERQUAD 2006 and refer to random errors only. The constants for the hydrolytic Zn(II) species were used in these calculations. The distribution and competition diagrams were computed with the HYSS program.^27^

### Spectroscopy

The absorption spectra were recorded on Varian Cary 300 Bio spectrophotometer, in the range 200–800 nm, using a quartz cuvette with an optical path of 1 cm. circular dichroism (CD) spectra were recorded on a Jasco J-1500 CD spectrometer in the 200–800 nm range, using a quartz cuvette with an optical path of 1 cm or with a cuvette with an optical path of 0.01 cm in the wavelength range 180–300 nm. The solutions were prepared in a water solution of 4 mM HClO_4_ at ionic strength *I* = 40 mM (SDS). The concentrations of solutions used for spectroscopic studies were similar to those in the potentiometric experiments; the Cu(II):ligand ratio was also 1:1. The UV–vis and CD spectroscopic parameters were calculated from the spectra obtained at the pH values corresponding to the maximum concentration of each particular species based on distribution diagrams.

### ROS (Reactive Oxygen Spieces) Generation

Experiments were based on the procedure described in article.^28^ Measurements were carried out on a Cary 60 spectrophotometer (Agilent Technologies) in the 200–800 nm range using a Hellma quartz cuvette with an optical path of 1 cm. Spectrophotometer was equipped with Single Cell Peltier Accessory (Agilent Technologies) to achieve a constant temperature (37 °C). Experiments were performed in the 50 mM Bis-Tris buffer (pH 7.4) in the presence of 200 μM piscidin complex, 0.5 mM H_2_O_2_, and 20 μM reporting molecule *N*,*N*-dimethyl-*p*-nitrosoaniline (NDMA) as a scavenger for the hydroxyl radical (OH^•^). Bleaching of the NDMA characteristic band (λ = 440 nm) suggests the production of assayed radicals.

### Liposome Preparation

Fluorescence spectra were recorded in a kinetic mode on a Varian Cary Eclipse spectrofluorometer using quartz fluorescence cuvettes with an optical path of 1 cm.

Ten mg of lipids (DOPC/DOPA:Cholesterol 30:30:40% mol %) was dissolved in 1 mL of chloroform and then dried under a stream of air for 4 h. The dry lipid film was hydrated with a solution of 6-carboxyfluorescein in HEPES buffer (20 mM 6-carboxyfluorescein, 10 mM HEPES, 150 mM NaCl, pH = 7.4), sonicated, and subjected to three freeze–thaw cycles (−15 to 60 °C).

To obtain homogeneous liposomes, the liposomal suspension was extruded 70 times through the polycarbonate membranes with a pore size 100 nm, mounted on Avanti Mini-Extruder (Avanti Polar Lipids). To remove untrapped 6-carboxyfluorescein, the liposomal suspension was filtered twice through a Sephadex-G50 column.

Five μM solutions of peptides in HEPES buffer were prepared. Each sample contained 1980 μL of peptide solution and 20 μL of liposomes. To obtain 100% of dye leakage, after each measurement 60 μL of 1% Triton X100 was added to the sample. 1980 μL of HEPES buffer and 20 μL of liposomes were measured to check the passive dye leakage. Each experiment was carried out for 15 min with excitation and emission wavelengths of 492 and 512 nm, respectively.

To assess the percent of disrupted liposomes, results were analyzed with the following formula
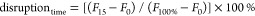
where *F*_15_—the intensity of fluorescence after 15 min, *F*_0_—the intensity of fluorescence in *t* = 0 s, and *F*_100%_—the intensity of fluorescence after Triton X100 addition.^29,30^

### DFT Calculations

Computational methods of theoretical chemistry were used to predict structure and stability of the ligands and complexes.^31−37^ Molecular orbital studies on Cu(II) and Zn(II) cations 1:1 complexes with piscidins 1, 2, and 3 ligands were done on the DFT level of theory. The starting structure of the peptide for DFT calculations was generated on the basis of the amino acid sequence in the preliminary β-strain secondary structures. After ligand folding (75 ps dynamics at 300 K, without cutoffs) using BIO + implementation of CHARMM force field, ligands were introduced to DFT optimizations as starting structures of the ligands. The starting metal cation positions in the complexes were arbitrarily chosen in the probable areas of the strong metal binding. Full optimizations of the complexes have been performed at the DFT level of theory. DFT calculations were performed with Gaussian 16 C.01^38^ suite of programs using the ωB97X-D^39^ long-range corrected hybrid density functional with damped atom–atom dispersion corrections were used with a double-ζ 6-31G(d,p) basis set containing polarization functions.

### Antimicrobial Activity Assay of Peptide and Peptide–Metal Ion Complex System

Seven reference strains from ATCC collection [*Acinetobacter baumannii* (ATCC 19606), *Klebsiella pneumoniae* (ATCC 700603), *Pseudomonas aeruginosa* (ATCC 27853), *Escherichia coli* (ATCC 25922), *Staphylococcus aureus* MRSA (ATTC 43300), *Enterococcus faecalis* (ATCC 29212), and *Candida albicans* (ATCC 10231)] were used for antimicrobial activity assay. Moreover, the clinical strains were also analyzed [MRSA (HD27 and HD33), *E. coli* (HD25, HD29, Ped76, and Ped117), *K. pneumoniae* (HD80, HD91, Ped91, and Ped112), and *A. baumannii* (HD47, HD82, HD90, and Ped64)].

The antimicrobial effect of analyzed peptides/complexes was performed according to the standard protocol using microdilution method with spectrophotometric measurement (λ = 580 nm at starting point and after 24 h) according to the ISO standard 20776-1:2019, ISO standard 16256:2021 and modified Richard’s method.^40−43^ Stock peptide solutions were prepared in deionized sterile water four times concentrated. Serial dilutions of the ligand/complex solution were made on 96-well microplates in the range between 0.5 and 256 μg/mL (for reference strains) or 0.5 and 32 μg/mL (for clinical strains). Tryptone Soya Agar plates were inoculated with microbial strains from glycerol stocks. After 24 h/37 °C incubation (for bacteria) or 24 h/25 °C (for fungus), a proper density of bacterial and fungal suspension was prepared using a densitometer [final inoculum (5 × 10^5^ CFU/mL for bacteria and 0.5–2.5 × 10^5^ CFU/mL for fungus) was prepared in Tryptic Soy Broth (TSB)]. Positive (TSB + strain) and negative controls (TSB) were also included in the test. Spectrophotometric solubility control of each peptide and the peptide–metal ion system was also performed. For each strain, the validation process was performed using the following antibacterial/antifungal agents: levofloxacin, gentamicin, and amphotericin B according to the EUCAST examination. The minimum inhibitory concentration (MIC) was determined as the lowest concentration of an antimicrobial agent that decreased the measured microbial growth to 50% as referred to the positive control. Obtained MIC values were for *A. baumannii* (ATCC 19606): levofloxacin 0.5 μg/mL, *E. coli* (ATCC 25922): gentamicin 4 μg/mL, *E. faecalis* (ATCC 29212): levofloxacin 4 μg/mL, *P. aeruginosa* (ATCC 27853): levofloxacin 1 μg/mL, *S. aureus* MRSA (ATCC 43300): levofloxacin 1 μg/mL, *K. pneumoniae* (ATCC 700603): gentamicin 4 μg/mL, and *C. albicans* (ATCC 10231): amphotericin B 1 μg/mL. Microplates were incubated at 37 ± 1 °C or 25 ± 1 °C for 24 h on the shaker (500 rpm). After this, the spectrophotometric measurement was performed at 580 nm, and then 50 μL aliquots of 1% (*m*/*v*) 2,3,5- triphenyltetrazolium chloride (TTC) solution were added into each well. TTC is a chemical indicator that is converted into red formazan crystals in living microbial cells. The minimum bactericidal concentration (MBC) or the minimum fungicidal concentration can be observed as the lowest concentration required to kill a particular microbial strain, determined by visual analysis after 24 h incubation with TTC (did not change the color to pink).

### Neutral Red Uptake Assay

For each peptide and peptide–metal ion system, where the antimicrobial activity was determined, a NR uptake assay was performed using human primary renal proximal tubule epithelial cells (RPTEC) from the ECACC collection. The experiment was performed according to ISO 10993–5:2009 and ISO/IEC 17025:2017 guidelines.^44,45^ A standard protocol for the NR assay was used from Nature Protocol.^46^ MEMα supplemented with 10% fetal bovine serum, 2 mM l-glutamine, and a suitable amount of antibiotics (amphotericin B and gentamicin) was used for the experiment. Prior to the assay, the cells (10^5^ cells/mL) were incubated for 24 h to promote adhesion. Stock peptide and peptide–metal solutions were prepared in deionized sterile water and then diluted 100 times in the medium. Also Cu(II) and Zn(II) salt solutions were checked to eliminate the potential cytotoxic effect of metal ions. After adding proper mixtures of testing compounds into each well, plates were incubated for 48 and 72 h in 5% CO_2_ at 37 °C. Next, the medium was removed and 100 μL of NR solution (40 μg/mL) was added to each well followed by incubation for 2 h at 37 °C. After removing the dye, wells were rinsed with PBS and left to dry. Then, NR destain solution (1% glacial acetic acid, 50% of 96% ethanol, and 49% of deionized water; v/v) was added to each well. The plates were shaken (30 min, 500 rpm) until NR was extracted from the cells and formed a homogeneous solution. The absorbance was measured using a microplate reader at 540 nm. As a negative control, untreated cells were considered as 100% of potential cellular growth. Furthermore, cells incubated with 1 μM staurosporine were used as the positive control.

## Results

### Stoichiometry

To determine the stoichiometry of the Zn(II) and Cu(II) complexes with piscidins, mass spectrometry was used.

Comparison of the experimental and simulated isotopic patterns confirms the presence of Zn(II) and Cu(II) complexes with piscidins 1–3 (Figures S1B,D,F and S2B,D,F). In the studied conditions, all complexes are observed only in a mononuclear form with a ligand/metal ratio of 1:1. Please keep in mind that in the scope of this work, only 1 to 1 metal to ligand ratio was studied, and judging by the number of histidines present in the sequence, it is quite probable that in the presence of metal excess, the ligands would bind more metal ion equivalents.

### Ligand Protonation

For piscidin 1, six deprotonation constants were detected. The first deprotonation constant refers to the lysine side chain (p*K*_a_ = 10.02). The second form, H_2_L with p*K*_a_ value 8.36 corresponds to deprotonation of N-terminal amine residue. The last four constants (p*K*_a_ values of 7.68, 7.74, 6.90, and 4.57), come from the deprotonation of four imidazole groups from the histidine side chains (Table 1). It is important to note that although potentiometry is a very precise method for the determination of ligand deprotonation constants and metal complex stability constants, it provides only macroconstants that cannot be assigned to individual deprotonating moieties. To determine such individual deprotonation constants (microconstants), typically NMR could be helpful (especially in cases when sequential overlap does not hinder doing so).

**Table 1:** Protonation and Stability Constants; Errors are Given in Parentheses

	piscidin 1 FFHHIF NH_2_-GIVHVGKTIHRLVTG		piscidin 2 FFHHIFRGIVHVGKTIHKLVTG		piscidin 3 FIHHIFRGIVHAGRSIGRFLTG	
	log β	log K	log β	log K	log β	log K
H_7_L	45.27 (5)	4.57 (His)	58.17 (8)	4.94 (His)	38.69 (5)	4.62 (His)
H_6_L	40.70 (3)	6.90 (His)	53.23 (6)	7.27 (His)	34.07 (3)	7.37 (His)
H_5_L	33.80 (2)	7.74 (His)	45.96 (5)	7.68 (His)	26.70 (2)	7.83 (His)
H_4_L	26.06 (3)	7.68 (His)	38.28 (5)	7.76 (His)	18.87 (3)	8.26 (N-term)
H_3_L	18.38 (2)	8.36 (N-term)	30.52 (5)	8.82 (N-term)	10.61 (1)	10.61 (Arg)
H_2_L	10.02 (1)	10.02 (Lys)	21.70 (3)	10.54 (Lys)		
HL			11.16 (5)	11.16 (Lys)		
Zn(II) complexes						
ZnH_4_L			42.52 (10)			
ZnH_3_L	29.97 (8)		35.85 (3)	6.67 (His)		
ZnH_2_L	23.37 (2)	6.60 (His)	27.81 (4)	8.04 (N-term)	24.22 (2)	
ZnHL	15.49 (2)	7.88 (N-term)	18.82 (5)	8.99 (H_2_O)	16.93 (2)	7.29 (N-term)
ZnL	6.32 (2)	9.17 (H_2_O)			8.00 (4)	8.93 H_2_O
ZnH_–1_L	–3.77 (9)	10.09 (Lys)	–0.57 (5)		–1.47 (4)	9.47 (Arg)
ZnH_–2_L					–11.73 (5)	10.26 (Arg)
ZnH_–3_L					–22.90 (5)	11.17 (Arg)
Cu(II) complexes						
CuH_5_L			51.59 (2)			
CuH_4_L						
CuH_3_L	33.30 (4)		40.22 (2)			
CuH_2_L	27.24 (4)	6.07 (His)	33.35 (3)	6.87 (His)	26.91 (3)	
CuHL	20.44 (5)	6.80 (His)	26.44 (3)	6.91 (His)		
CuL	13.15 (6)	7.28 (His)	16.81 (5)	9.63 (Lys)	13.82 (4)	
CuH_–1_L	3.33 (10)	9.82 (Lys)			6.35 (7)	7.47 (His)
CuH_–2_L	–7.27 (9)	10.60 (Arg)	–4.89 (6)		–3.78 (12)	10.13 (Arg)
CuH_–3_L					–14.50 (10)	10.72 (Arg)

Piscidin 2 differs from piscidin 1 only by substitution of Arg18 to Lys18. The first two values indicate the deprotonation of the two lysine residues (p*K*_a_ of 11.16 and 10.54). The third constant comes from the N-terminal amine group (p*K*_a_ = 8.82). Similarly, to the previous peptide, the last four deprotonation constants are related to deprotonation of the four imidazole groups from histidines (p*K*_a_ equal 7.76, 7.68, 7.27, and 4.94).

For piscidin 3, five deprotonation constants were determined. The subsequent p*K*_a_ values of 10.61 and 8.26 are derived from the deprotonated Arg residue and N-terminal amine group, respectively. The last three (p*K*_a_ = 7.83, 7.37, and 4.62) are associated with histidine side chains deprotonation. The Arg p*K*_a_ value is relatively low as for a guanidinum group; however, depending on the chemical environment around it, its value can be several orders of magnitude lower in certain sequences and easily fall in the measuring scope of the electrode.^47^

### Zn(II) Complexes

In the case of piscidin 1, five species are observed (Table 1 and Figure S3A). The first, ZnH_3_L, starts to form at around pH 6.00. Then, probably three imidazole groups coordinate the metal ion. The next complex form, ZnH_2_L, dominates in solution in the pH range 6.70–7.80. The large difference between the p*K*_a_ values of the complex (p*K*_a_ = 6.60) and free peptide (7.68) indicates coordination of the fourth histidine residue. At the same time, on the basis of the determined stability of the complexes (discussed in the following sections of this work), it is known that piscidins form {3N_im_} coordination type complexes with zinc(II). Therefore, it is assumed that in the case of the ZnH_2_L form, another histidyl residue may be coordinated with the simultaneous detachment of one of the previously bound imidazole nitrogens or these two forms exist in equilibrium. The third form, ZnHL with a p*K*_a_ value of 7.88, appears at pH around 7.00 and achieves its maximum around 8.50. Most likely, in these species, deprotonation of the nonbinding N-terminal amine group occurs. The next constant, p*K*_a_ = 9.17, corresponds to the ZnL form in which, presumably, the deprotonation of a water molecule coordinated to the Zn(II) ion occurs. The last species, ZnH_–1_L is observed at pH above 9.00. Its p*K*_a_ value (10.09) suggests deprotonation of the lysine residue, which does not coordinate with the metal ion.

For piscidin 2, five complex forms were detected (Table 1 and Figure S3B). The first complex, ZnH_4_L, with a log β of 42.52 forms around pH 6.50 and most likely involves three imidazole nitrogens in Zn(II) coordination. The next form, ZnH_3_L with a p*K*_a_ value of 6.67, dominates in solution in the pH range 7.00–8.00 and is probably formed as a result of coordination of zinc(II) by the next histidyl residue (similar to piscidin 1). Subsequently, the ZnH_2_L form appears at pH around 7.30 and is the most abundant around pH 8.50. In this species, deprotonation of the nonbonding N-terminal amine group occurs. In the case of the fourth complex, ZnHL with p*K*_a_ = 8.99, the deprotonation of a bound water molecule is observed. This complex forms around pH 8.30 and dominates in the pH range 9.00–9.80. The last complex form, ZnH_–1_L appears at pH around 9.30 and dominates in solution above pH 9.80.

At pH range 2–11, piscidin 3 forms six complex species with Zn(II) (Table 1 and Figure S3C). The first one observed at acidic pH, ZnH_2_L, is formed around pH 6.00 and is the most abundant around pH 6.95. As in the case of piscidins 1 and 2, the Zn(II) ion is probably coordinated by three imidazole nitrogens derived from histidyl residues. The next complex, ZnHL with p*K*_a_ = 7.29 refers to deprotonation of nonbonding N-terminal amine group. This form dominates in the solution at pH range 7.30–8.90. The deprotonation of a water molecule bound to the central Zn(II) atom results in the formation of the ZnL complex, with a p*K*_a_ value of 8.93. This form reaches its maximum around pH 9.20. The subsequent three stability constants (p*K*_a_ = 9.47, 10.26, and 11.17) correspond to the deprotonation of the three nonbonding arginine residues.

### Cu(II) Complexes

All analyzed piscidins contain a specific structural motif (called ATCUN), where the free N-terminal amine group and histidine residue in the third position are present. This motif enables the formation of highly stable, square-planar complexes with Cu(II) and Ni(II) ions.^48^ Since all analyzed peptides interact with Cu(II) in a similar way, piscidin 1 is described below as a representative example.

For Cu(II)-piscidin 1, in the pH range 2.00–11.00, six complex species are observed. Copper starts to coordinate at pH around 4.8, forming the CuH_3_L complex, which achieves its maximum at pH 5.60 (Table 1 and Figure S4A). In the UV–vis spectrum (Figure S5A), at pH 5.00 and 517 nm, a minor peak can be observed. Additionally, the intensity of this peak increases significantly at pH 6.00, indicating the sudden formation of the 4N complex between pH 5.00 and 6.00 (in the pH range 2–4 no clear peaks are detected). Moreover, in the CD spectrum (Figure S6A), at pH 6.00, two characteristic peaks at 487 and 564 nm confirm the presence of a square-planar complex (Figures S5A and S6A).^49^ The situation when 4N square-planar complex forms rapidly is typical for Cu(II) complexes with the ATCUN motif. The next three species—CuH_2_L, CuHL, and CuL—with p*K*_a_ values 6.07, 6.80, and 7.28, respectively, correspond to deprotonation of the three nonbinding histidine residues. The last two forms, CuH_–1_L and CuH_–2_L with stability constants 9.82 and 10.60, respectively, indicate the deprotonation of the nonbonding lysine and arginine residue.

Spectroscopic data and distribution diagrams for Cu(II)-piscidin 2 and Cu(II)-piscidin 3 complexes are presented in Figures S4B,C, S5B,C, and S6B,C.

### DFT Calculations

**Figure 2:**
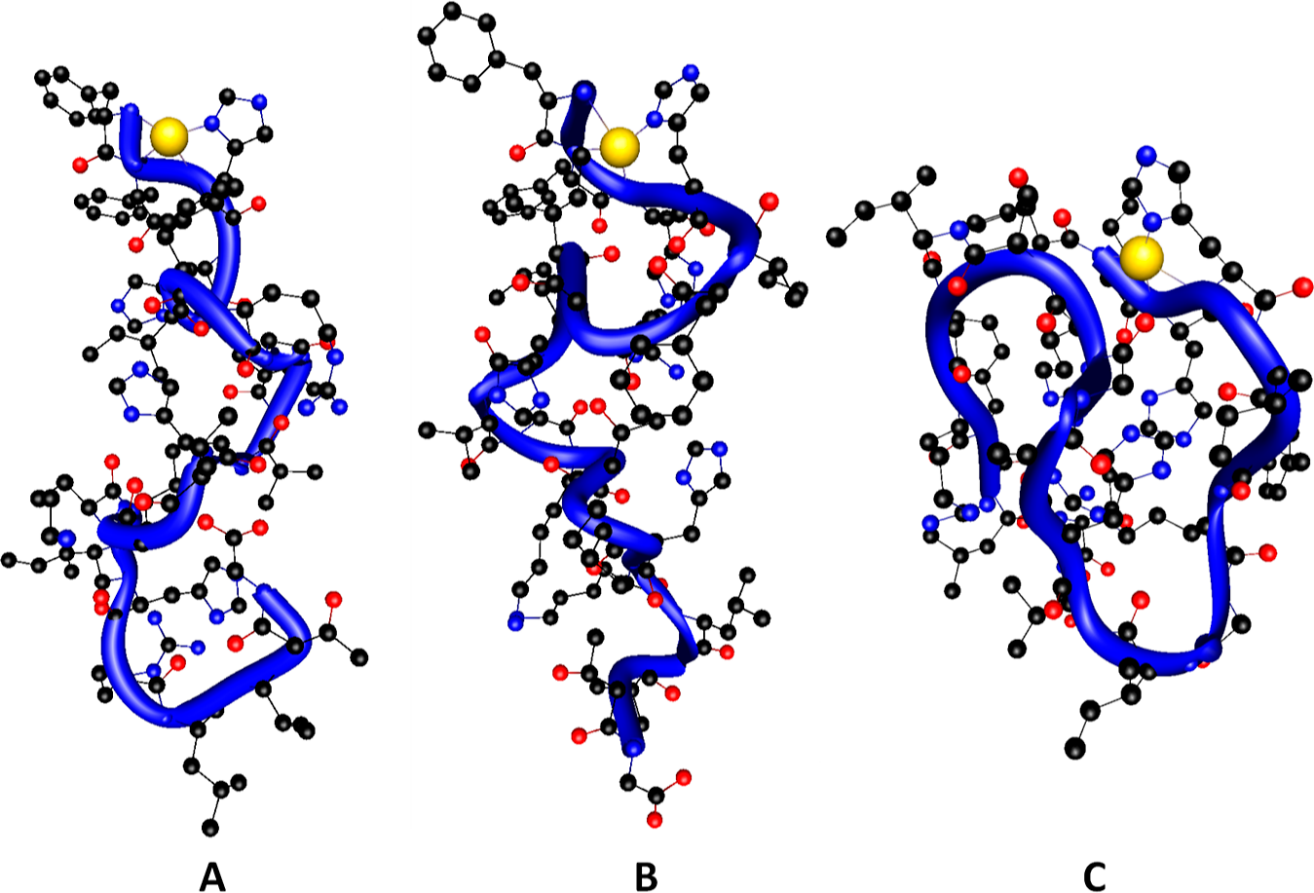
Structures of complexes between piscidins 1, 2, 3, and Cu(II) cation calculated at the DFT level of theory; blue tubes follow backbones.

**Figure 3:**
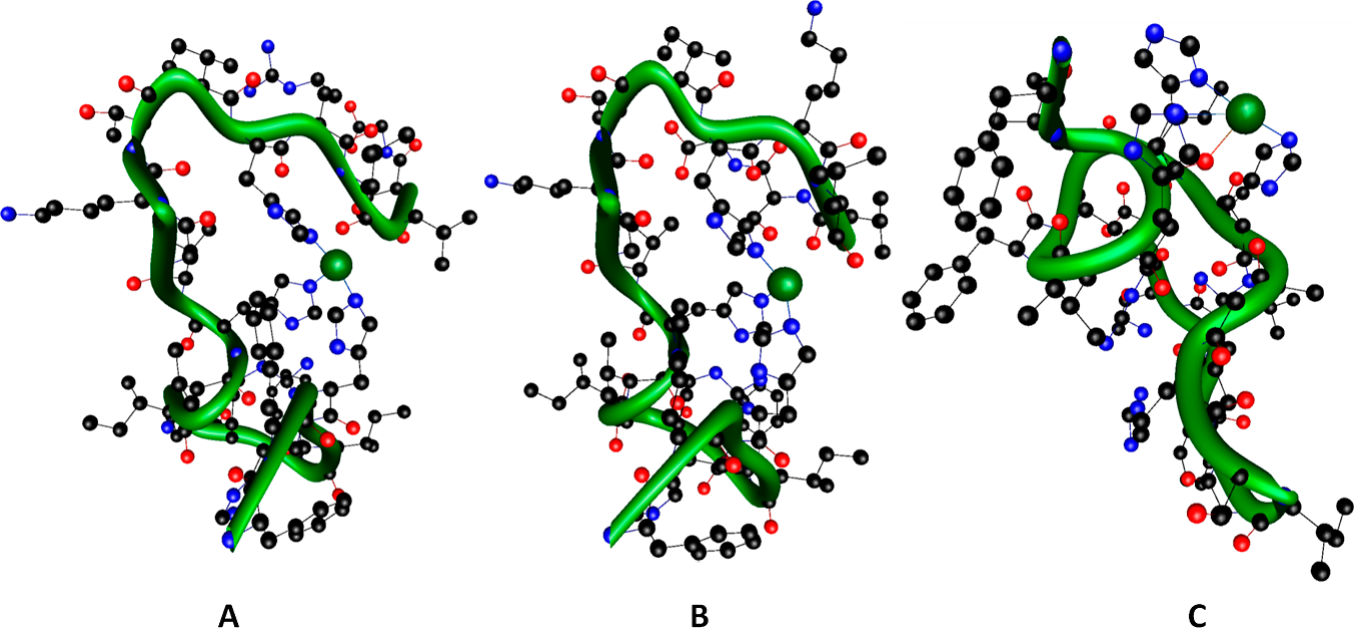
Structures of complexes between piscidins 1, 2, 3, and Zn(II) cation calculated at the DFT level of theory, green tubes follow backbones.

At the DFT level of theory, we observe three multiple connected complexes with each Cu(II) and Zn(II) ions (Figures 2 and 3).

For Cu(II) complexes, DFT results are in perfect agreement with potentiometric and spectroscopic data and show that all three Cu(II) complexes are formed by coordination of four nitrogens from the ATCUN motif: the N-terminal amine group and two subsequent amide groups from the peptide backbone and from the His3 imidazole ring (Figure 2).

In each complex, the Cu(II)–NH_2_ bonds are definitely longer than the other ones. Cu(II)–N_im_ interactions are the strongest among all observed, thus forming the shortest bonds (Table 2).

**Table 2:** Metal–Ligand Distances in Angstroms [Å] for Piscidins 1, 2, and 3 and Cu(II) Complexes from the DFT Calculations

	piscidin 1	piscidin 2	piscidin 3
NH_2_	2.238	2.589	2.501
amide 1	1.965	1.933	1.935
amide 2	2.142	2.010	1.982
His3 (imidazole)	1.861	1.926	1.908

In contrast to the complexes with copper(II), zinc(II) complexes form two different patterns of interactions.

Piscidins 1 and 2 form a 3N type complex, where His4, His11, and His17 imidazole rings are engaged. Piscidin 3 also forms a 3N type complex, but due to the lack of His17 residue, it uses different binding sites; in this case, His3, His4, and His11 take part in coordination. Additionally, a supporting C=O···Zn(II) interaction with the carbonyl oxygen from His4 was observed (Table 3 and Figure 3). The probable cause of this interaction is Phe/Ile substitution at position 2. The Phe2 residue in piscidins 1 and 2 contains a bulky, rigid side chain that impedes the coordination of His3 imidazole nitrogen. Ile2, present in piscidin 3, has a more flexible side chain than Phe2, which allows for a proper spatial arrangement of the peptide. Thus, both the His3 imidazole nitrogen and His4 carbonyl oxygen could take part in zinc(II) binding.

**Table 3:** Metal–Ligand Distances in Angstroms [Å] for Piscidins 1, 2, 3, and Zn(II) Complexes from the DFT Calculations

	piscidin 1	piscidin 2	piscidin 3
His3			2.206
His4	2.163	1.979	2.006
His11	1.984	1.982	2.186
His17	2.165	2.199	
C=O (His4)			2.101

### Comparison of Metal Binding Affinities

On the basis of potentiometric measurements, competition diagrams were prepared. They show a hypothetical situation when equimolar amounts of reagents are mixed, enabling a comparison of the affinity of peptides for a given metal ion.

For both the Zn(II) and Cu(II) piscidin complexes, the observed affinities are fairly comparable. In the case of Zn(II) complexes, at physiological pH Zn(II)-piscidin 3 is slightly more stable than the other two complexes (Figure S7A), which is probably due to the additional interaction of Zn(II) with the carbonyl group located at the His4 residue. This interaction occurs only with piscidin 3 and may be due to differences in the peptide sequence at position 2. The Ile2 residue present in piscidin 3 has a more flexible side chain compared to the Phe2 residue present in piscidins 1 and 2. As a result, piscidin 3 can obtain a better spatial fit, allowing the carbonyl group of His4 to be engaged.

For Cu(II) complexes, piscidin 2 shows a moderately higher affinity toward Cu(II) ions than piscidin 3 and piscidin 1 (Figure S7B). Similar stability of the complexes results from similarities in Cu(II) coordination by piscidins 1, 2, and 3.

### Antimicrobial Activity and Cytoxicity

Antimicrobial activity of piscidins and their metal complexes was determined on reference strains of two Gram-positive (*E. faecalis* and methicillin-resistant *S. aureus*), four Gram-negative bacteria (*E. coli*, *K. pneumoniae*, *A. baumannii*, and *P. aeruginosa*), and one yeast species (*C. albicans*). Moreover, clinical strains of *A. baumannii*, *E. coli*, MRSA, and *K. pneumoniae* were also tested.

All examined peptides, both with and without the metal ions, show excellent antimicrobial activity, with MIC values even lower than those of some of the currently used drugs such as fosfomycin or nitrofurantoin (Tables S1, 4, and 5).

**Table 4:** Results of Antimicrobial Activity Tests of Piscidins 1–3 and Their Complexes with Zn(II) and Cu(II) against Reference Strains, Expressed as MIC/MBC Values [μg/mL]; n/d-No Action Detected^a^

strain	piscidin 1		Cu(II)-piscidin 1		Zn(II)-piscidin 1		piscidin 2		Cu(II)-piscidin 2		Zn(II)-piscidin 2		piscidin 3		Cu(II)-piscidin3		Zn(II)-piscidin3	
	MIC	MBC	MIC	MBC	MIC	MBC	MIC	MBC	MIC	MBC	MIC	MBC	MIC	MBC	MIC	MBC	MIC	MBC
*E. coli*	8	8	8	8	4	4	8	8	16	16	8	8	32	32	16	16	32	32
*E. faecalis*	32	32	32	32	32	32	32	32	64	64	32	32	32	256	32	32	32	64
*K. pneumoniae*	16	16	32	32	16	16	16	16	64	64	16	16	n/d	n/d	64	64	128	128
*S. aureus* MRSA	16	16	16	16	16	16	16	32	32	32	32	32	64	64	32	32	32	32
*A. baumannii*	4	4	4	4	4	4	4	4	8	8	4	4	8	8	4	4	8	8
*P. aeruginosa*	64	64	64	128	64	64	64	64	128	128	64	64	256	256	128	128	n/d	n/d
*C. albicans*	64	64	64	64	64	64	64	64	64	64	64	64	64	128	64	64	64	128

a The experiments were carried out in accordance with the requirements of ISO 20776–1:2019 and ISO 16256:2021.^40,41^ Values marked in green indicate reduced MIC/MBC values of complexes against peptides (higher antimicrobial activity), red – MIC/MBC values of complexes higher than MIC/MBC of peptides (lower antimicrobial activity).

**Table 5:** Results of Antimicrobial Activity Tests of Piscidins 1–3 and Their Complexes with Zn(II) and Cu(II) against Clinical Strains, Expressed as MIC/MBC Values [μg/mL]; n/d-No Action Detected^a^

strain		piscidin 1		Cu(II)- piscidin 1		Zn(II)-piscidin 1		piscidin 2		Cu(II)- piscidin 2		Zn(II)- piscidin 2		piscidin 3		Cu(II)- piscidin 3		Zn(II)- piscidin 3	
		MIC	MBC	MIC	MBC	MIC	MBC	MIC	MBC	MIC	MBC	MIC	MBC	MIC	MBC	MIC	MBC	MIC	MBC
*A. baumannii*	HD47	4	4	4	4	4	4	4	4	8	8	4	4	8	8	8	8	8	8
	HD82	16	16	32	32	16	16	16	16	32	32	16	16	n/d	n/d	n/d	n/d	n/d	n/d
	HD90	8	8	8	8	4	4	4	4	8	8	4	4	8	8	8	8	16	16
	Ped64	16	16	32	32	16	16	8	8	16	16	16	16	n/d	n/d	n/d	n/d	n/d	n/d
*E. coli*	HD25	8	8	16	16	8	8	16	16	32	32	16	16	n/d	n/d	16	16	n/d	n/d
	HD29	16	16	32	32	16	16	16	16	32	32	16	16	n/d	n/d	n/d	n/d	n/d	n/d
	Ped76	8	8	8	8	4	4	8	8	8	8	8	8	n/d	n/d	16	16	32	32
	Ped117	8	8	8	8	8	8	8	8	8	8	8	8	32	32	16	16	16	16
MRSA	HD27	32	32	32	32	32	32	32	32	n/d	n/d	32	32	n/d	n/d	n/d	n/d	n/d	n/d
	HD33	8	8	16	16	8	8	16	16	32	32	16	16	n/d	n/d	32	32	n/d	n/d
*K. pneumoniae*	Ped91	32	32	n/d	n/d	32	32	16	16	32	32	16	16	n/d	n/d	32	32	32	32
	Ped112	32	32	32	32	32	32	16	32	32	32	32	32	n/d	n/d	32	n/d	32	32
	HD80	8	8	8	8	8	8	4	4	4	4	4	4	16	16	4	4	16	16
	HD91	32	32	n/d	n/d	32	32	32	32	32	32	16	16	n/d	n/d	n/d	n/d	n/d	n/d

a The experiments were carried out in accordance with the requirements of ISO 20776-1:2019 and ISO 16256:2021.^40,41^ Values marked in green indicate reduced MIC/MBC values of complexes against peptides (higher antimicrobial activity), red—MIC/MBC values of complexes higher than MIC/MBC of peptides (lower antimicrobial activity).

Piscidins 1 and 2 show lower MIC values than piscidin 3 and are thus more efficient antimicrobial agents. Furthermore, an overall trend can be observed where, with some exceptions, the addition of Cu(II) ion slightly increases the MIC values for piscidins 1 and 2, which means a slight worsening of their antimicrobial activity. Oppositely, the addition of Cu(II) ions to piscidin 3 decreased MIC values (Tables 4 and 5). These observations are consistent with the results obtained for piscidins 1 and 3 and their Cu(II) complexes against planktonic bacteria, *Bacillus cereus*,^50^ where piscidin 1 kills 100% of the tested bacteria at 5 μM concentration, while piscidin 3 does so at 20 μM. Interestingly, metalation of piscidin 3 reduced the MIC value to 10 μM, improving the bacterial killing potency twofold. Also, after the metalation of piscidin 1, a slight increase in the percentage of surviving bacteria could be observed, suggesting an inhibitory effect of copper(II) on piscidin 1 antimicrobial activity.

On the other hand, the addition of Zn(II) ions to piscidins 1 and 2, in the vast majority of cases, does not cause significant changes between the MIC values of peptides and their Zn(II) complexes (Tables 4 and 5). A different trend is observed for piscidin 3, where metal coordination usually increases (or moderately increases) its antimicrobial activity.

The observed effect of metal ions on the MIC values of piscidins 1 and 2 is quite small, which suggests that the mechanism of antimicrobial activity of these peptides most likely does not depend solely on the presence of Zn(II) and Cu(II) ions. In contrast, Cu(II) and Zn(II) complexes with piscidin 3 have a higher antimicrobial potency than that of piscidin 3 alone. In order to obtain additional information on the potential mechanism of action of piscidins and their complexes, experiments detecting the formation of reactive oxygen species and potential membrane disrupting abilities were carried out.

In order to evaluate the cytotoxic potential of peptides and their metal complexes on a normal RPTEC cell line, the compounds were administered at concentrations equal to the MIC determined through antimicrobial activity assay against reference bacterial strains. The findings are summarized in Table 6. The microbial strains for which the MIC value was equal to or exceeded the cytotoxic concentration are also included in the last column. This occurrence was particularly noted in the case of *P. aeruginosa* and *C. albicans*, whereby limiting the utility of piscidins against these strains. In contrast, strains susceptible to piscidin eradication without apparent cytotoxicity include *A. baumannii* and *E. coli*.

**Table 6:** Cytotoxic Concentrations [μg/mL] of Piscidins 1–3 and Their Complexes with Zn(II) and Cu(II) Were Determined through the NR Uptake Assay [46] Using the RPTEC Cell Line^a^

compound	cytotoxic conc. [μg/mL]	excluded strains
piscidin 1	64	*P. aeruginosa*; *C. albicans*
Cu(II)-piscidin 1	32	*E. faecalis*; *K. pneumoniae*
		*P. aeruginosa*; *C. albicans*
Zn(II)-piscidin 1	64	*P. aeruginosa*; *C. albicans*
piscidin 2	64	*P. aeruginosa*; *C. albicans*
Cu(II)-piscidin 2	64	*E. faecalis*; *K. pneumoniae*
		*P. aeruginosa*; *C. albicans*
Zn(II)-piscidin 2	32	*E. faecalis*; *S. aureus*
		*P. aeruginosa*; *C. albicans*
piscidin 3	256	*K. pneumoniae*; *P. aeruginosa*
Cu(II)-piscidin 3	64	*K. pneumoniae*; *P. aeruginosa*
		*C. albicans*
Zn(II)-piscidin 3	64	*K. pneumoniae*; *P. aeruginosa*
		*C. albicans*

a In the last column, reference microbial strains are listed for which the cytotoxic concentration of a given compound equaled or exceeded the indicated MIC value.

### Membrane Disrupting Ability and ROS Formation

To assess the membrane disrupting ability of piscidins and their Zn(II) and Cu(II) complexes, experiments were carried out. Liposomes, imitating the bacterial membrane, were filled with a fluorescent dye (carboxyfluorescein), which leaked out after liposome damage. Leakage was monitored by the spectrofluorometric measurements and seen as an increasing intensity of fluorescence.

Figure S8A–C shows the ability of piscidins to damage liposomes and the influence of the Cu(II) and Zn(II) ions on this process. In all cases, it can be readily seen that the addition of copper(II) ions drastically increases the ability to disrupt the model membranes. Oppositely to Cu(II), the presence of Zn(II) ions impairs this process. Figure S8D compares the membranolytic properties of piscidins. Piscidin 3 is much less membrane-damaging than piscidins 1 and 2, suggesting a different mechanism of antimicrobial action, which is in agreement with previous works.^51^

In order to obtain additional information about the mechanism of action of piscidin metal complexes, experiments were carried out to detect the formation of reactive oxygen species.

No ROS are formed in the case of Zn(II) complexes, while for copper complexes, we observed a decreasing value of absorbance with time, indicating the formation of reactive oxygen species (hydroxyl radical) in solution (Figure S9). Results obtained for copper(II) complexes of piscidins 1 and 2 show high similarity and suggest the generation of a much larger amount of hydroxyl radical compared to the Cu(II)-piscidin 3 complex.

These findings may suggest that the mechanism of the antimicrobial activity of copper(II) complexes of piscidins 1 and 2 is at least partially related to the induction of oxidative damage.

## Conclusions

In this work, we analyzed Zn(II) and Cu(II) complexes of piscidins 1, 2, and 3, focusing on their thermodynamic properties and the influence of metal ion coordination on their antimicrobial activity.

All examined complexes exist in equimolar stoichiometry. All three piscidins coordinate Cu(II) in the same way, via their ATCUN motif, where the N-terminal amine group, two subsequent backbone nitrogens, and imidazole ring from His3 residue are engaged in formation of a square-planar, highly stable complex. For Zn(II)-peptide binding systems, two modes of coordination are present: piscidin 1 and 2 bind Zn(II) by imidazole nitrogens from His4, His11, and His17 and piscidin 3, due to the lack of His17 residue, coordinates Zn(II) by His3, His4, and His11 imidazole nitrogens and an additional supporting interaction, formed by the carbonyl oxygen from His4.

The correlation between metal binding and antimicrobial potency of piscidin complexes is far from being trivial. It is certain that both piscidins and their metal complexes have a very high antimicrobial activity. The obtained MIC values are often lower than those of currently used therapeutics such as nitrofurantoin or fosfomycin. Among the studied peptides, piscidin 3 shows slightly lower antimicrobial activity than piscidins 1 and 2. In the case of metal complexes, some general trends are observed: (i) the addition of Cu(II) ions generally slightly decreases the antimicrobial potency of piscidins 1 and 2, while (ii) in contrast, an increase of antimicrobial potency is observed for piscidin 3 after it coordinates with Cu(II); and (iii) no significant effect of Zn(II) binding to piscidins 1 and 2 on their biological activity is observed, while its coordination to piscidin 3 moderately enhances its antimicrobial potency.

The high antimicrobial activity of piscidin metal complexes is due neither to their stability nor to a change in the secondary structure (Figure S10), but the formation of free radicals and membrane damage is in some cases still an option—Cu(II) complexes with piscidins 1 and 2 can form hydroxyl radicals, which may probably be related to the antimicrobial activity of these species. These results correlate both with those of studies on model liposomes (that show a higher membrane disrupting activity of Cu(II) complexes of piscidins 1 and 2 than that of piscidin 3) and with literature data, also confirming the formation of reactive oxygen species by Cu(II)-piscidin complexes. According to the studies of Comert et al.,^52,53^ the radicals formed at that time may contribute to the formation of damage in the cell membrane of the pathogen, causing the breakdown of double bonds in the lipids present there. Clearly, a different mechanism (most likely an intercellular targeted one) is observed for Cu(II)-piscidin 3. Zn(II) coordination to piscidins 1 and 2 has vaguely any effect on their antimicrobial potency, showing that, with some exceptions, it is not really metal coordination but rather the intrinsic properties of piscidins themselves that make them antimicrobial.

On the other hand, in most cases, the coordination of Cu(II) and Zn(II) enhances the antimicrobial potency of piscidin 3, showing that not only piscidin 3 but also its metal complexes have a different mode of action than piscidins 1 and 2. The reasonably low MIC values of piscidins and their metal complexes make them potentially attractive for possible pharmaceutical applications.

## Supporting Information Available

ESI-MS spectra and MS spectra of Zn(II)-piscidin 1, Zn(II)-piscidin 2, and Zn(II)-piscidin 3 samples; MS spectra of Cu(II)-piscidin 1, Cu(II)-piscidin 2, and Cu(II)-piscidin 3 samples; distribution diagrams for the formation of Zn(II) complex with piscidin 1, Zn(II) complex with piscidin 2, and Zn(II) complex with piscidin 3; distribution diagrams for the formation of Cu(II) complex with piscidin 1, Cu(II) complex with piscidin 2, and Cu(II) complex with piscidin 3; UV–vis spectra of Cu(II) complexes with piscidin 1, piscidin 2, and piscidin 3 in pH range 2–11; CD spectra of Cu(II) complexes with piscidin 1, piscidin 2, and piscidin 3 in pH range 2–11; competition plot between piscidins 1, 2, 3, and Zn(II) or Cu(II), describing complex formation at different pH values in a hypothetical situation, in which equimolar amounts of all reagents are mixed; percentage of disrupted liposomes by piscidin 1 and its Zn(II) and Cu(II) complexes, piscidin 2 and its Zn(II) and Cu(II) complexes, and piscidin 3 and its Zn(II) and Cu(II) complexes; NDMA decomposition in the presence of 200 μM Cu(II)-piscidin complexes; secondary structure of piscidin 1 and its copper(II) and zinc(II) complexes; and examples of MIC breakpoints values from EUCAST Version 14.0 [1] for bacteria (PDF)

## Abbreviations

AMP: antimicrobial peptide
EMA: European Medicines Agency
FDA: U.S. Food and Drug Administration
MBC: minimum bactericidal concentration
MFC: minimum fungicidal concentration
MIC: minimum inhibitory concentration
NDMA: *N*,*N*-dimethyl-*p*-nitrosoaniline
RPTEC: human primary renal proximal tubule epithelial cells
TTC: triphenyltetrazolium chloride
