# Supplementary material for: Zn(II) and Cu(II) Coordination Enhances the Antimicrobial Activity of Piscidin 3, but Not That of Piscidins 1 and 2
Source: Inorg Chem. 2024 Jul 1;63(28):12958–68. doi: 10.1021/acs.inorgchem.4c01659 (PMC11256756; doi:10.1021/acs.inorgchem.4c01659)
Supplement: Supplementary file 1 — ic4c01659_si_001.pdf [file ic4c01659_si_001.pdf]

SUPPLEMENTARY INFORMATION

Zn(II) and Cu(II) coordination enhances the antimicrobial activity of piscidin 3, but not that of piscidin 1 and 2

Miller Adriana[a], Mikołajczyk Aleksandra[b], Bellotti Denise[c], Garstka Kinga[a], Wątły Joanna[a], Hecel Aleksandra[a], Wieczorek Robert[a], Matera-Witkiewicz Agnieszka[b], Rowińska-Żyrek Magdalena*[a]*

a) Faculty of Chemistry, University of Wroclaw, ul. F. Joliot-Curie 14, 50-383 Wroclaw, Poland

b) Screening of Biological Activity Assays and Collection of Biological Material Laboratory, Wroclaw Medical University Biobank, Faculty of Pharmacy, Wroclaw Medical University, ul. Borowska 211a, 50-556 Wroclaw, Poland

c) Department of Chemical, Pharmaceutical and Agricultural Sciences, University of Ferrara, Via Luigi Borsari 46, 44121 Ferrara, Italy

Figure S1 presents spectra for Zn(II) complexes of piscidins. In the Figure S1A spectrum for Zn(II)-piscidin 1 complex is shown, where six signals are assigned to the: piscidin 1 (m/z = 858.2, z = 3+), its sodium (m/z = 865.5, z = 3+) and potassium adduct (m/z = 870.8, z = 3+), Zn(II)-piscidin 1 complex (m/z = 878.5, z = 3+) and its sodium (m/z = 886.1, z = 3+) and potassium (m/z = 891.5, z = 3+) adduct.

In the Figure S1C a spectrum registered for the Zn(II)-piscidin 2 complex is shown. Two visible signals are assigned to the single ligand (m/z = 636.6, z = 4+) and Zn(II)-piscidin 2 complex (m/z = 652.1, z = 4+).

Figure S1E shows spectrum for Zn(II)-piscidin 3 complex. Two main signals come from the single peptide (m/z = 623.6, z = 4+) and potassium adduct of Zn(II)-piscidin 3 complex (m/z = 648.1, z = 4+).

Figure S2 presents the results of the measurements for Cu(II) complexes of piscidins. In the Figure S2A, a spectrum for the Cu(II)-piscidin 1 can be observed, with six main peaks visible.

They are identified as a single peptide (*m/z* = 858.2, *z* = 3+), its sodium (*m/z* = 865.5, *z* = 3+) and potassium adduct (*m/z* = 870.8, *z* = 3+), Cu(II)-piscidin 1 complex (*m/z* = 878.8, *z* = 3+) and its sodium (*m/z* = 886.1, *z* = 3+) and potassium adduct (*m/z* = 891.4, *z* = 3+).

Figure S2C shows spectrum for the Cu(II)-piscidin 2 complex, where two visible peaks are assigned to the single ligand (*m/z* = 848.5, *z* = 3+) and complex (*m/z* = 868.8, *z* = 3+).

In the Figure S2E, spectrum for the Cu(II)-piscidin 3 complex can be found, where three main signals are assigned to the Cu(II)-piscidin 3 complex (*m/z* = 852.1, *z* = 3+) and its sodium (*m/z* = 859.4, *z* = 3+) and potassium adduct (*m/z* = 864.8, *z* = 3+).

A)

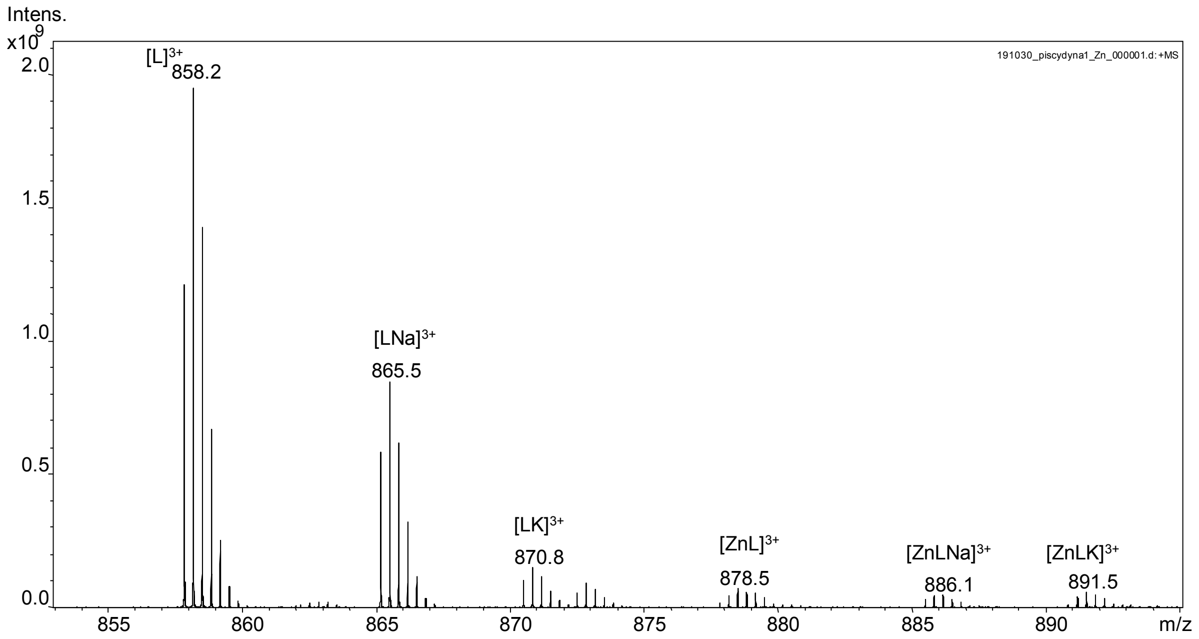

Intens.
x10^9
191030_piscydyna1_Zn_000001.d: +MS
[L]3+
858.2
[LNa]3+
865.5
[LK]3+
870.8
[ZnL]3+
878.5
[ZnLNa]3+
886.1
[ZnLK]3+
891.5
m/z

B)

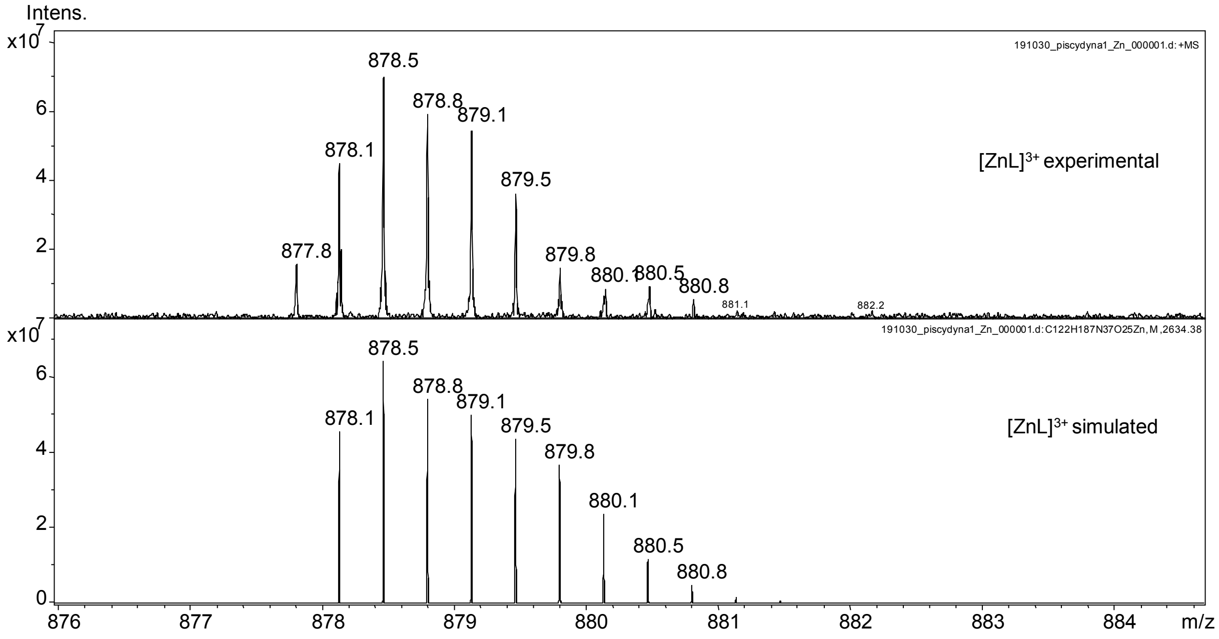

Intens.
x10^7
191030_piscydyna1_Zn_000001.d: +MS
[ZnL]3+ experimental
877.8
878.1
878.5
878.8
879.1
879.5
879.8
880.1
880.5
880.8
191030_piscydyna1_Zn_000001.d: C122H187N37O25Zn, M ,2634.38
[ZnL]3+ simulated
m/z

C)

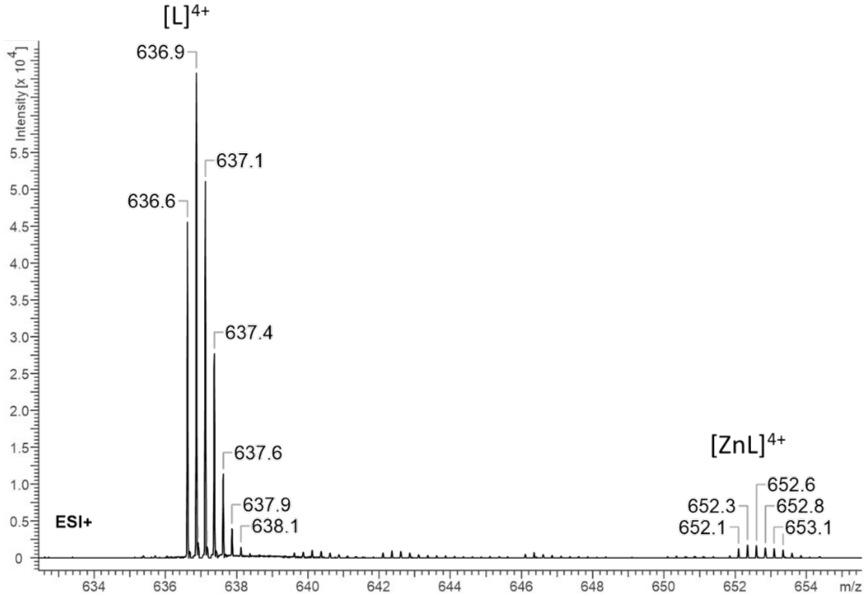
[L]4+
636.9
637.1
636.6
637.4
637.6
637.9
638.1
[ZnL]4+
652.6
652.3
652.8
652.1
653.1
ESI+
Intensity [x 10^4]
m/z

D)

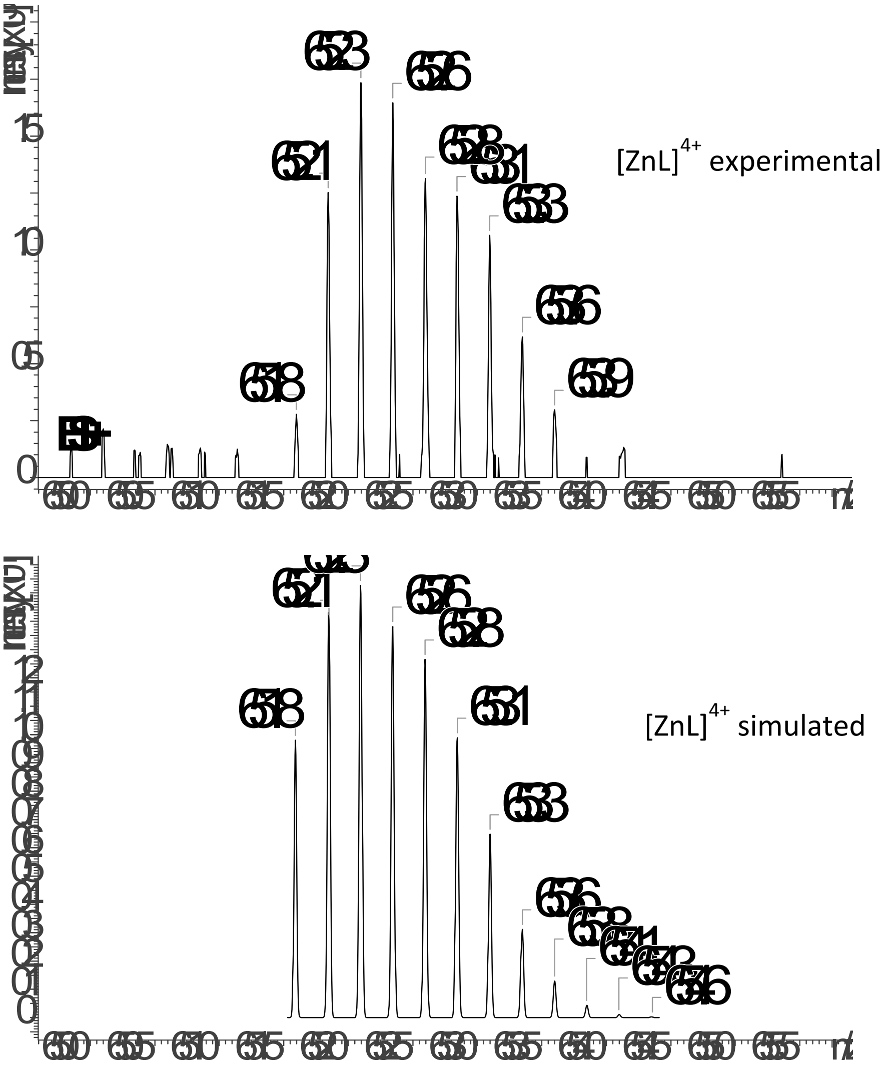
[ZnL]4+ experimental
[ZnL]4+ simulated

E)

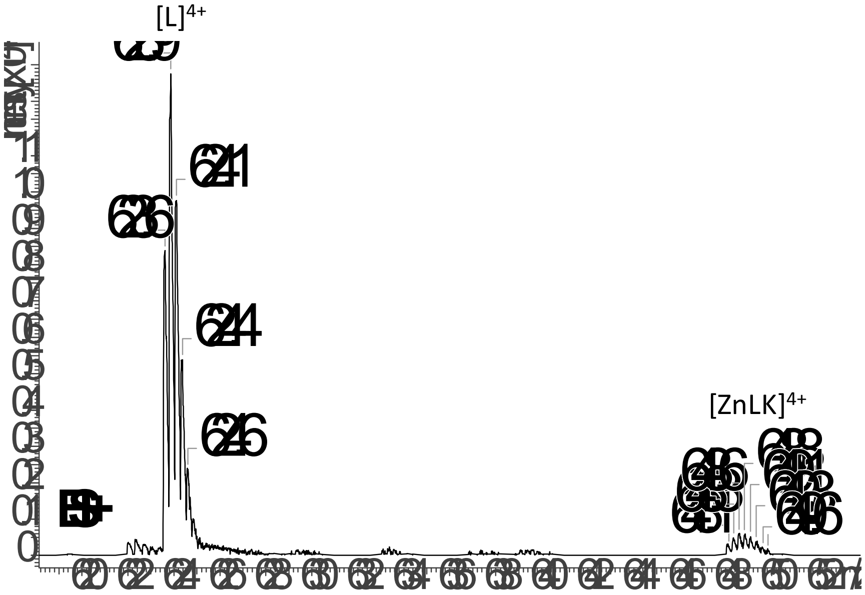

F)

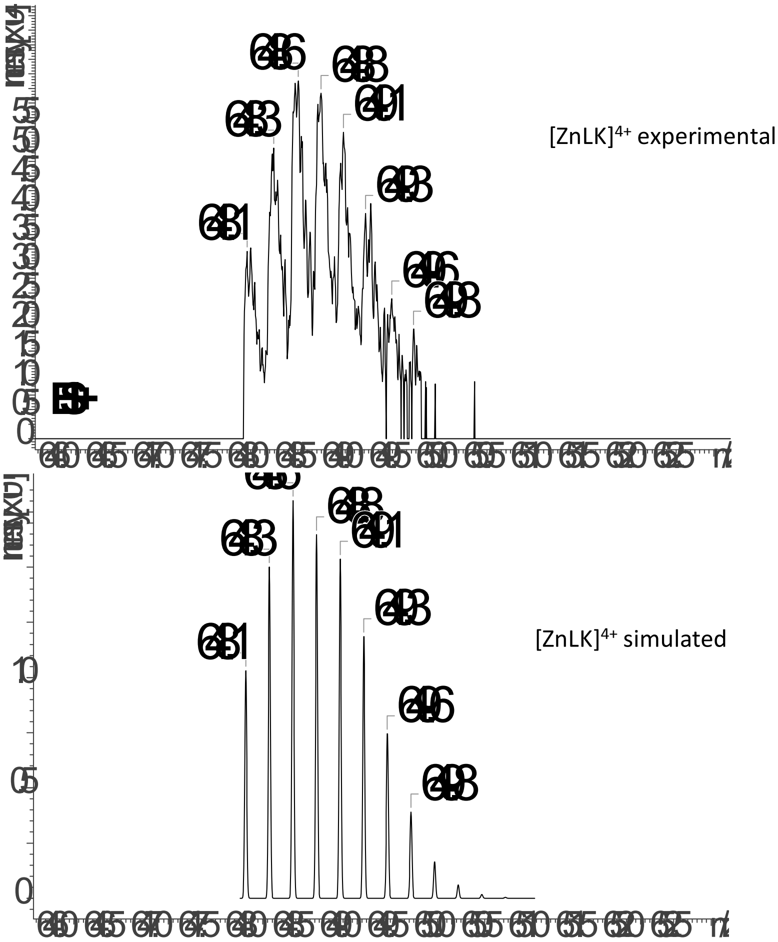

Fig. S1: ESI-MS spectra of: MS spectra of Zn(II)-piscidin 1 (A, B), Zn(II)-piscidin 2 (C, D) and Zn(II)-piscidin 3 (E, F) samples. In the B, D and F spectra, the experimental (top) and simulated (bottom) results are compared to clearly show the presence of the complex. Conditions: [Zn(II)] = [piscidin 1] = [piscidin 2] = [piscidin 3] = 3 x 10^{-4} M in a 1:1 methanol-water mixture; M^{2+}:peptide ratio was 1:1, pH = 6

A)

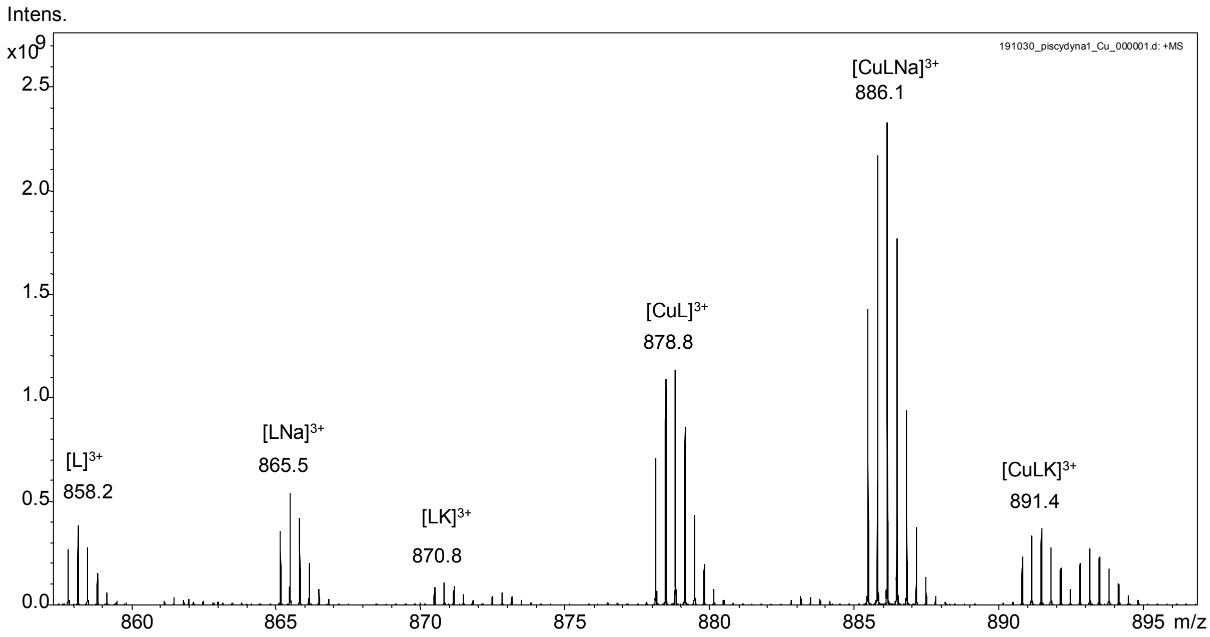

B)

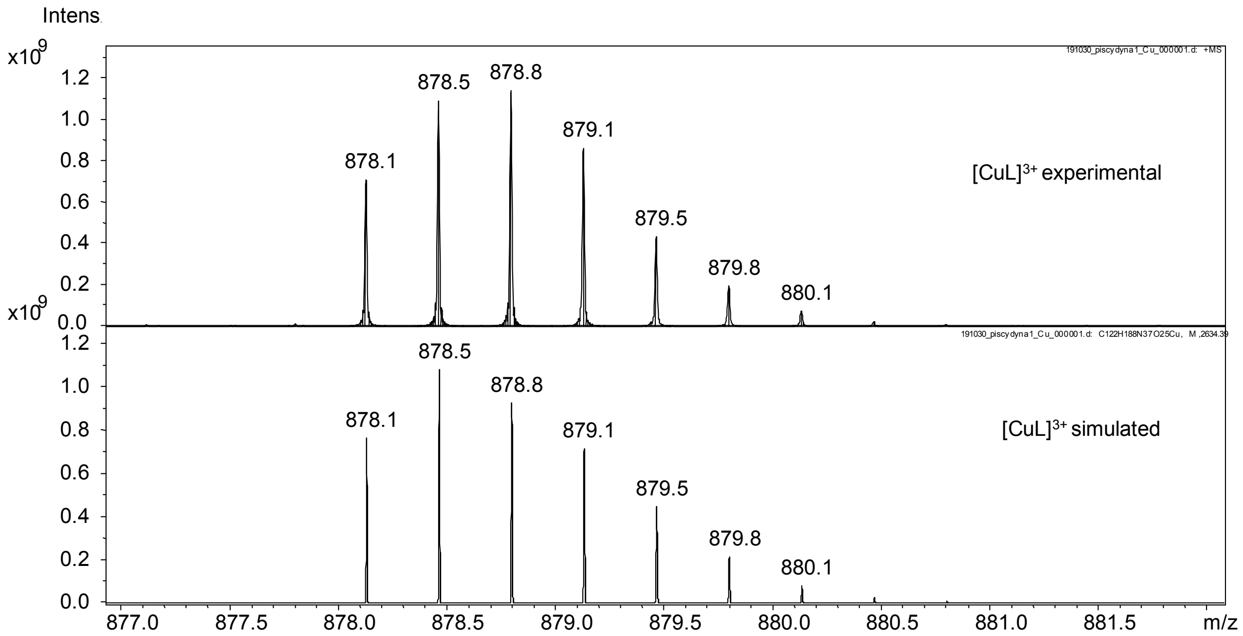
Intens.
x10^9
191030_piscydyna1_Cu_000001.d: +MS
878.1
878.5
878.8
879.1
879.5
879.8
880.1
[CuL]3+ experimental
x10^9
191030_piscydyna1_Cu_000001.d: C122H188N37O25Cu, M ,2634.39
878.1
878.5
878.8
879.1
879.5
879.8
880.1
[CuL]3+ simulated
877.0 877.5 878.0 878.5 879.0 879.5 880.0 880.5 881.0 881.5
m/z

C)

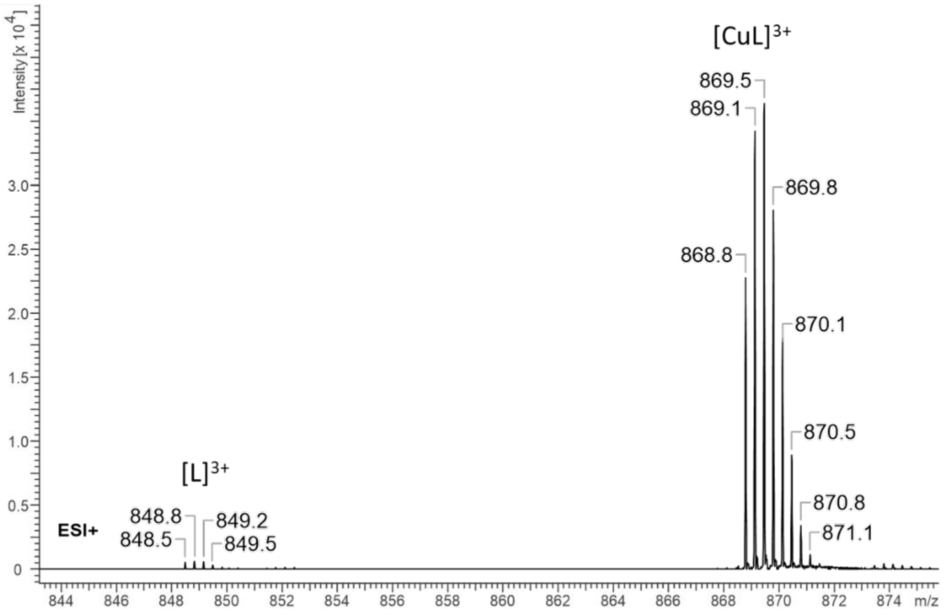

[CuL]3+
869.5
869.1
869.8
868.8
870.1
870.5
870.8
871.1
[L]3+
848.8
849.2
848.5
849.5
ESI+
Intensity [x 10^4]
m/z

D)

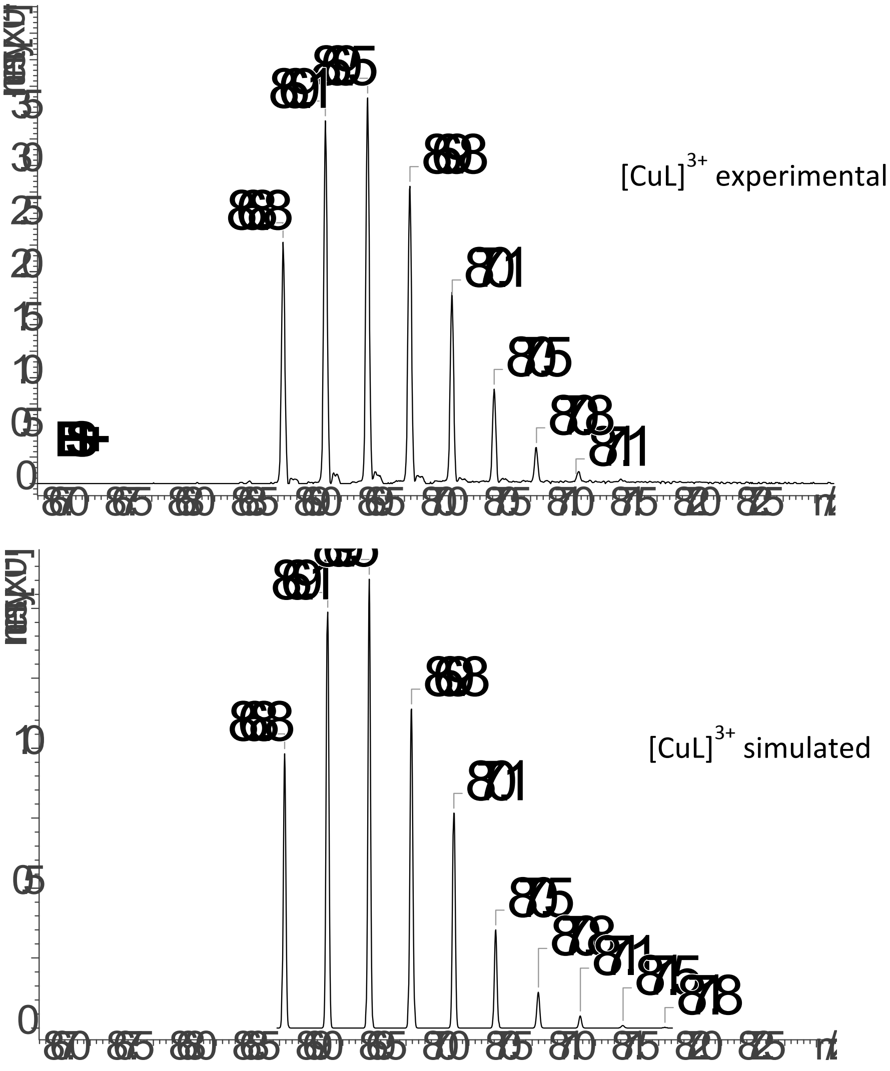

[CuL]3+ experimental
[CuL]3+ simulated

E)

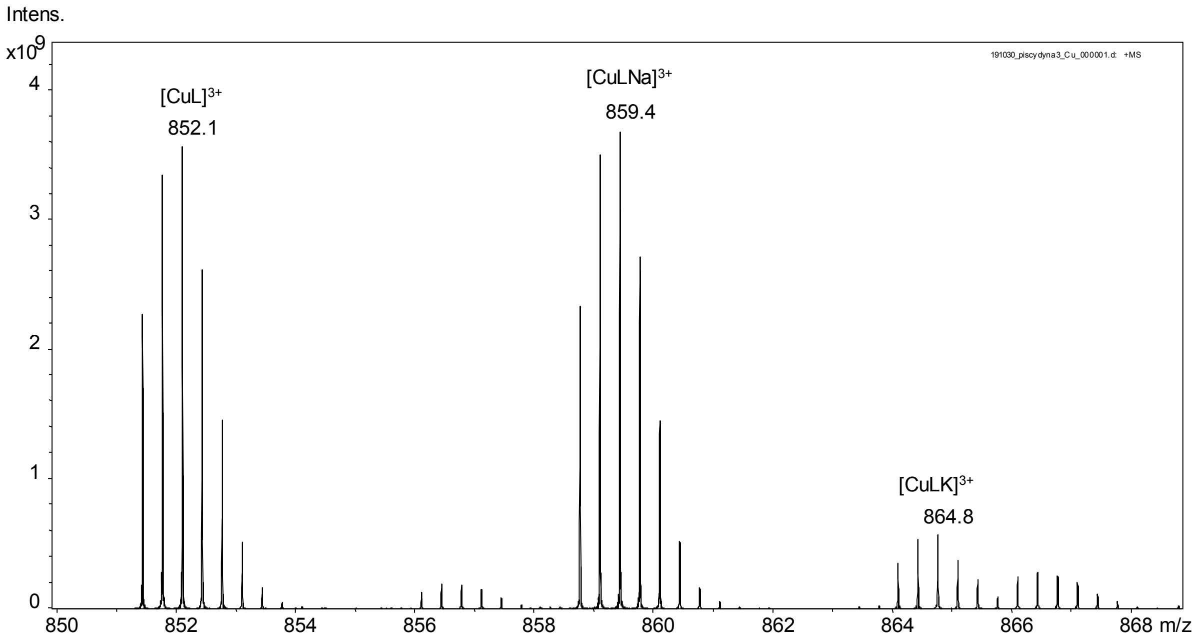

F)

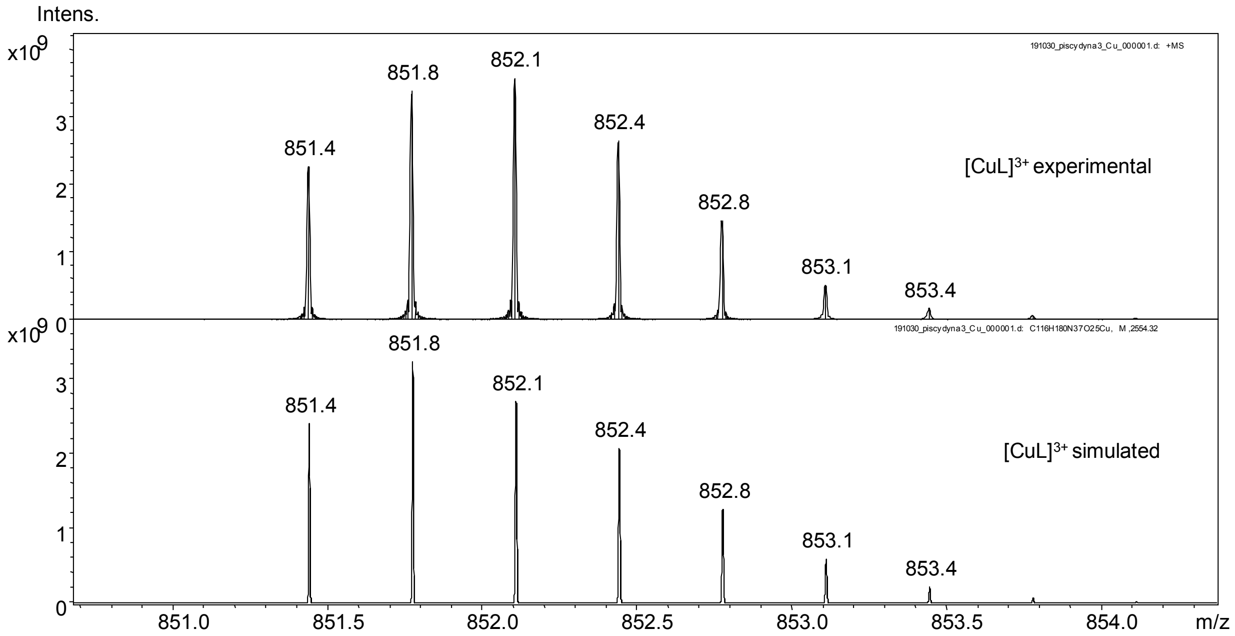

Fig. S2: MS spectra of Cu(II)-piscidin 1 (A, B), Cu(II)-piscidin 2 (C, D) and Cu(II)-piscidin 3 (E, F) samples. In the B, D and F spectra, the experimental (top) and simulated (bottom) results are

compared to clearly show the presence of the complex. Conditions: [Cu(II)] = [piscidin 1] = [piscidin 2] = [piscidin 3] = 3 x 10^{-4} M in a 1:1 methanol-water mixture; M^{2+}:peptide ratio was 1:1, pH = 6

A)

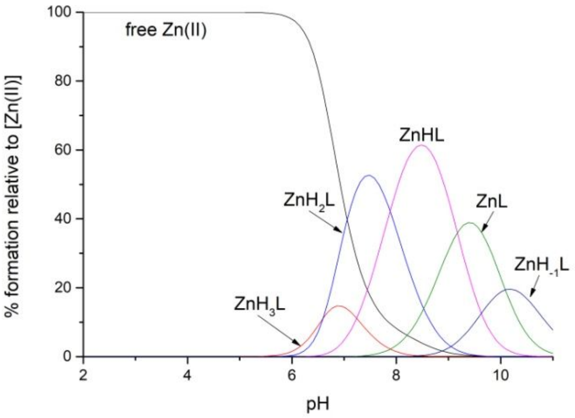
free Zn(II)
ZnHL
ZnH_2L
ZnL
$ZnH_{-1}L$
ZnH_3L
% formation relative to [Zn(II)]
pH

B)

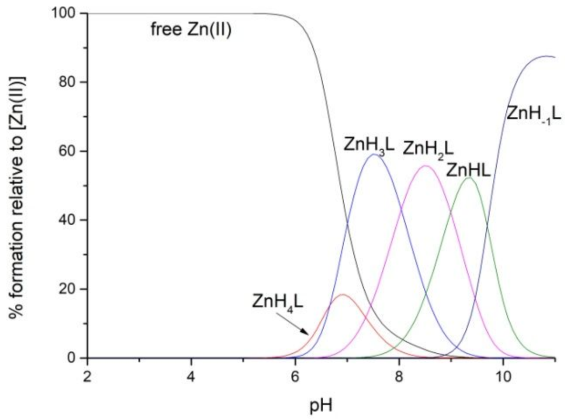
free Zn(II)
$ZnH_{-1}L$
ZnH_3L
ZnH_2L
ZnHL
ZnH_4L
% formation relative to [Zn(II)]
pH

C)

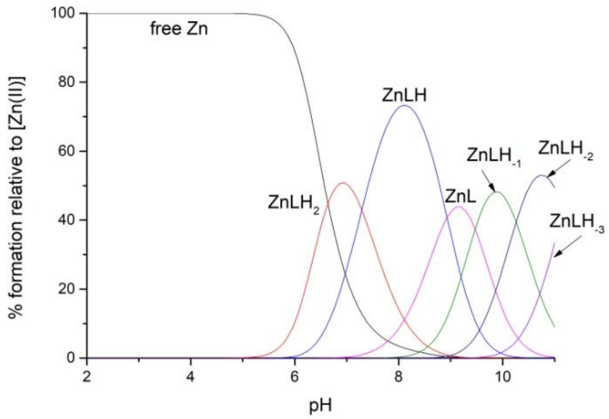
free Zn
ZnLH
$ZnLH_{-1}$
$ZnLH_{-2}$
ZnL
$ZnLH_2$
$ZnLH_{-3}$
% formation relative to [Zn(II)]
pH

Fig. S3: Distribution diagrams for the formation of: A) Zn(II) complex with piscidin 1; B) Zn(II) complex with piscidin 2; C) Zn(II) complex with piscidin 3; T=298 K, I= 40 mM SDS, $[M^{2+}]=0.5\cdot10^{-3}$ M; M^{2+}:L molar ratio = 1:1

A)

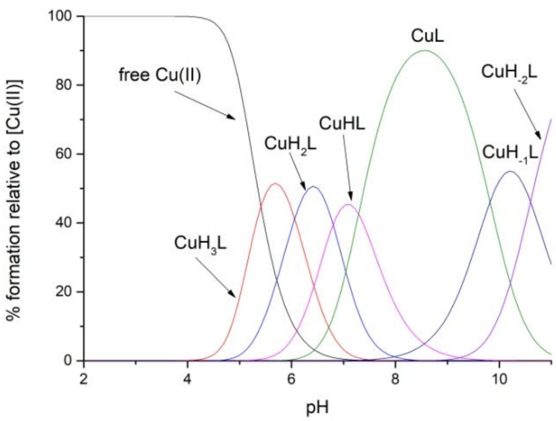

B)

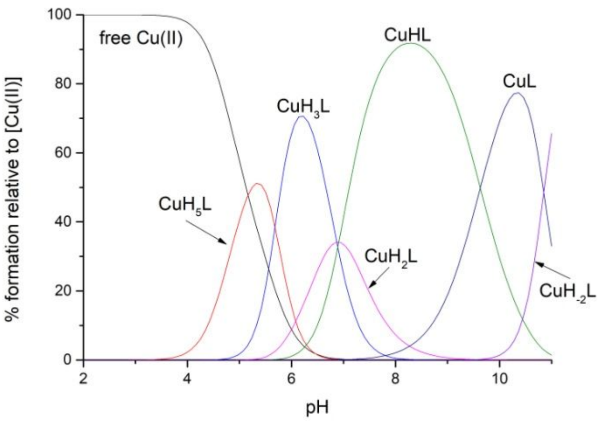

C)

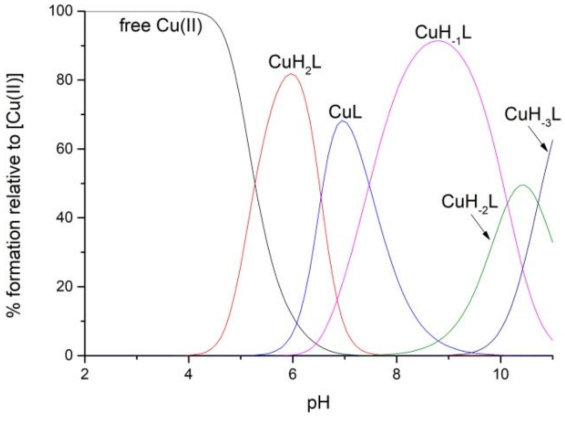

Fig. S4: Distribution diagrams for the formation of: A) Cu(II) complex with piscidin 1; B) Cu(II) complex with piscidin 2; C) Cu(II) complex with piscidin 3; T=298 K, I= 40 mM SDS, $[M^{2+}]=0.5\cdot10^{-3}$ M; M^{2+}:L molar ratio = 1:1

A)

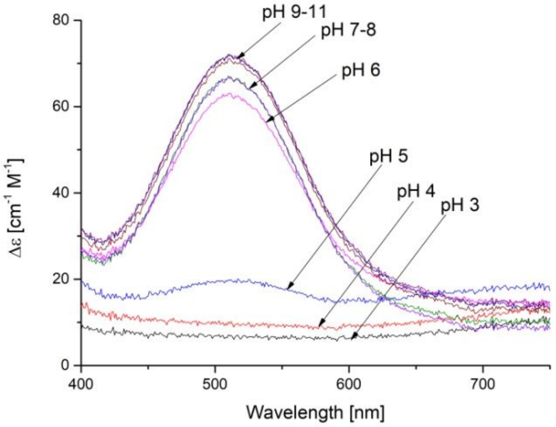

B)

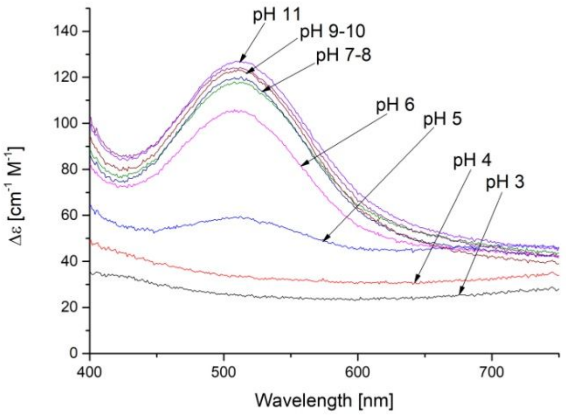

C)

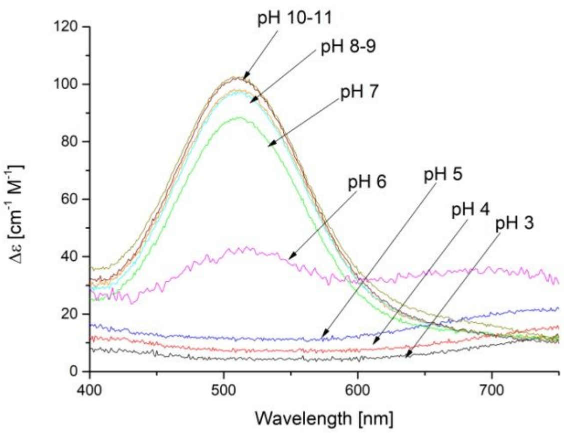

Fig. S5: UV-Vis spectra of Cu(II) complexes with: A) piscidin 1; B) piscidin 2; C) piscidin 3 in pH range 2-11. Conditions: T = 298 K, I = 40 mM SDS, [Cu(II)] = [piscidin 1] = [piscidin 2] = [piscidin 3] = 0.001 M.

A)

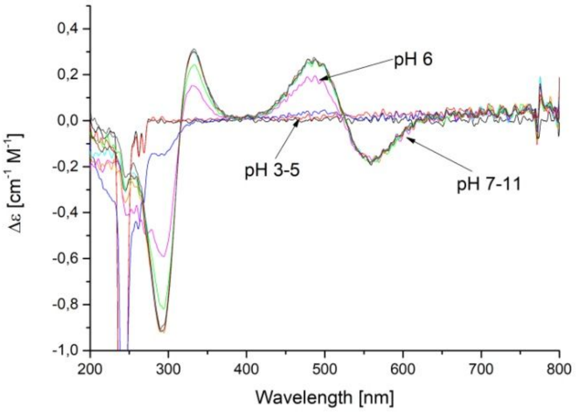

B)

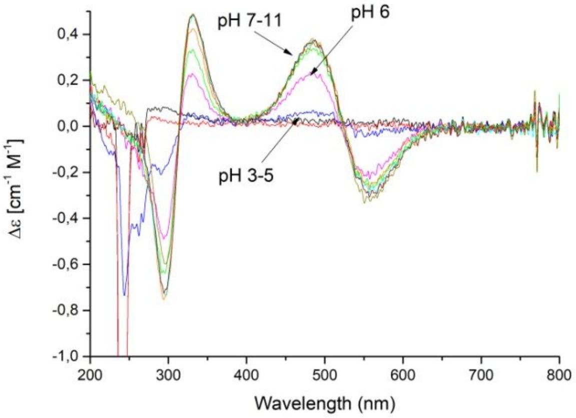

C)

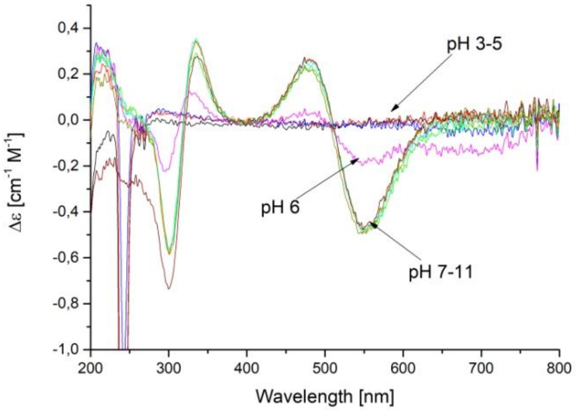

Fig. S6: CD spectra of Cu(II) complexes with: A) piscidin 1; B) piscidin 2; C) piscidin 3 in pH range 2-11. Conditions: T = 298 K, I = 40 mM SDS, [Cu(II)] = [piscidin 1] = [piscidin 2] = [piscidin 3] = 0.001 M.

A)

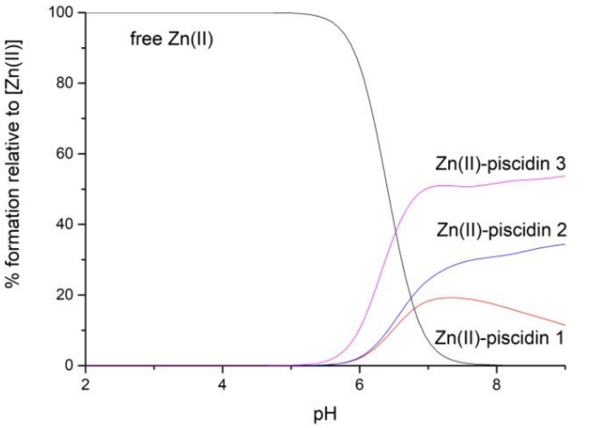

B)

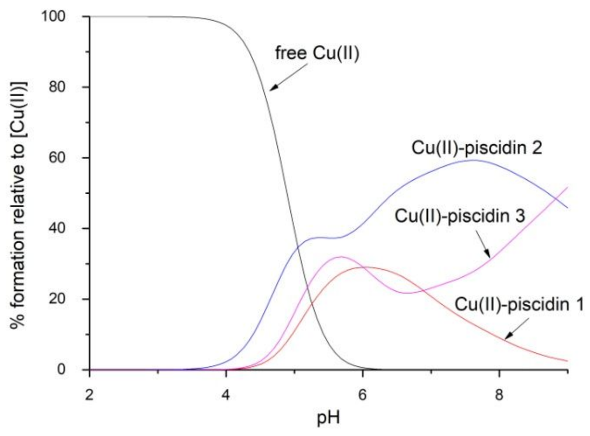

Fig. S7: Competition plot between piscidin 1, 2, 3 and Zn(II) (A) or Cu(II) (B), describing complex formation at different pH values in a hypothetical situation, in which equimolar amounts of all reagents are mixed.

A)

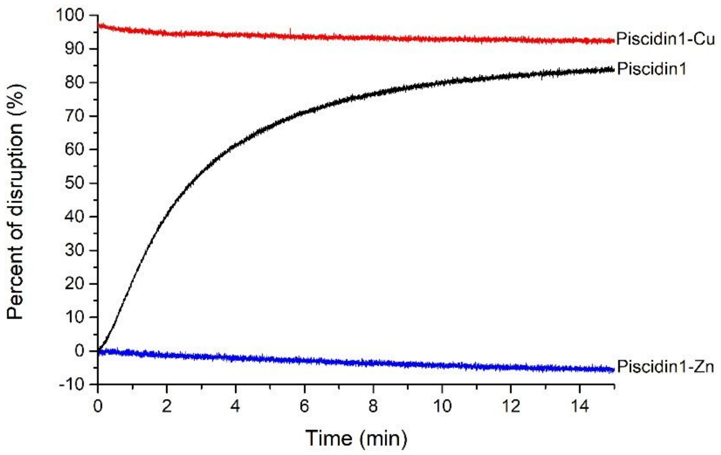
Percent of disruption (%)
Time (min)
Piscidin1-Cu
Piscidin1
Piscidin1-Zn

B)

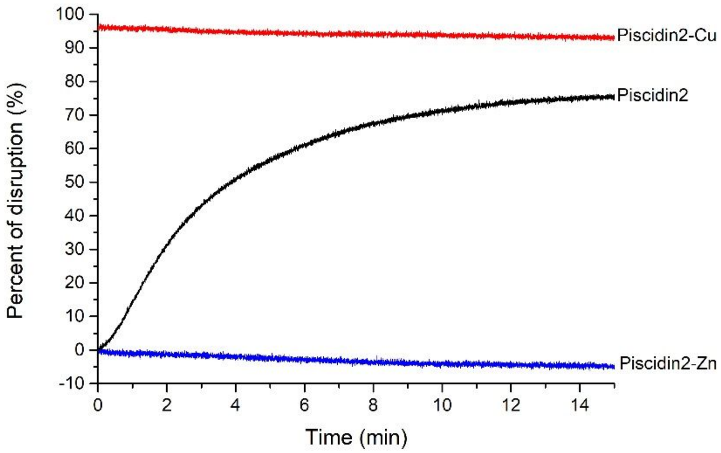
Percent of disruption (%)
Time (min)
Piscidin2-Cu
Piscidin2
Piscidin2-Zn

C)

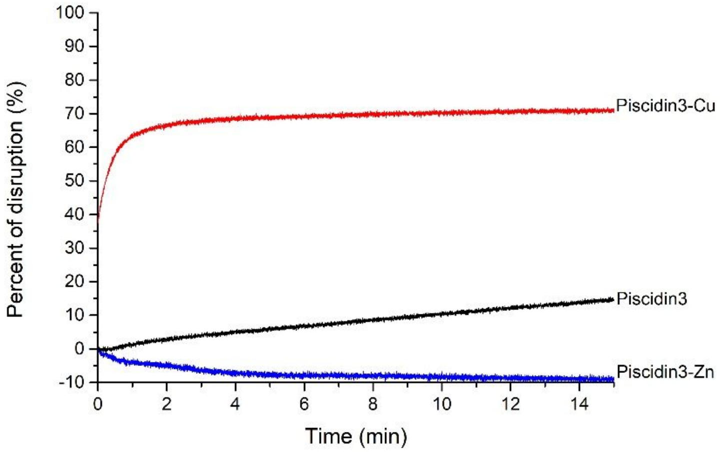
Percent of disruption (%)
Time (min)
Piscidin3-Cu
Piscidin3
Piscidin3-Zn

D)

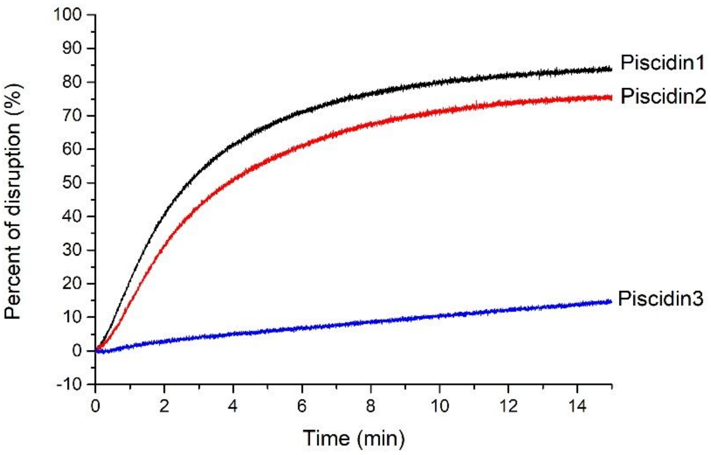

Fig. S8: Percentage of disrupted liposomes by: A) piscidin 1 and its Zn(II) and Cu(II) complexes; B) piscidin 2 and its Zn(II) and Cu(II) complexes; C) piscidin 3 and its Zn(II) and Cu(II) complexes. Diagram D) presents comparison of all tested piscidins. All samples were prepared in HEPES buffer (10 mM HEPES, 150 mM NaCl, pH = 7.4). Conditions: T = 298 K, [Cu(II)] = [Zn(II)] = [piscidin 1] = [piscidin 2] = [piscidin 3] = 5 μM

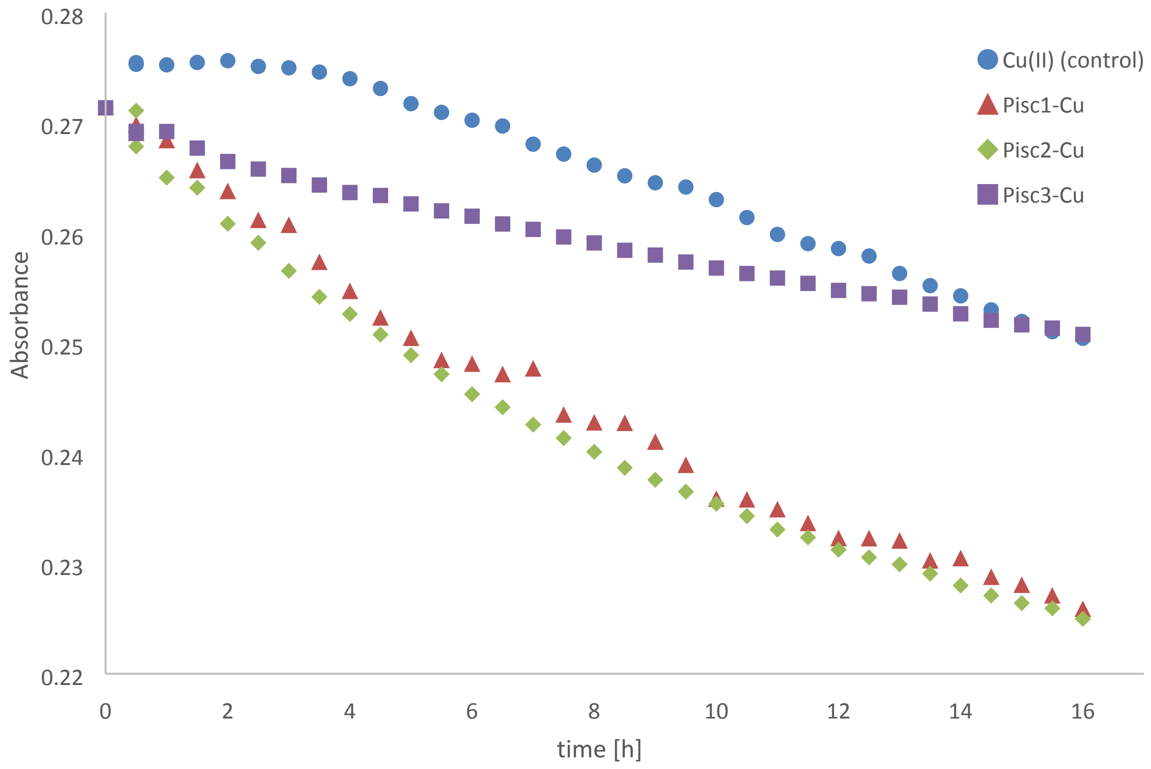

Fig. S9: NDMA decomposition in the presence of 200%µM Cu(II)-piscidin complexes. Decreasing absorbance at λ=440 nm indicates to the formation of hydroxyl radical.

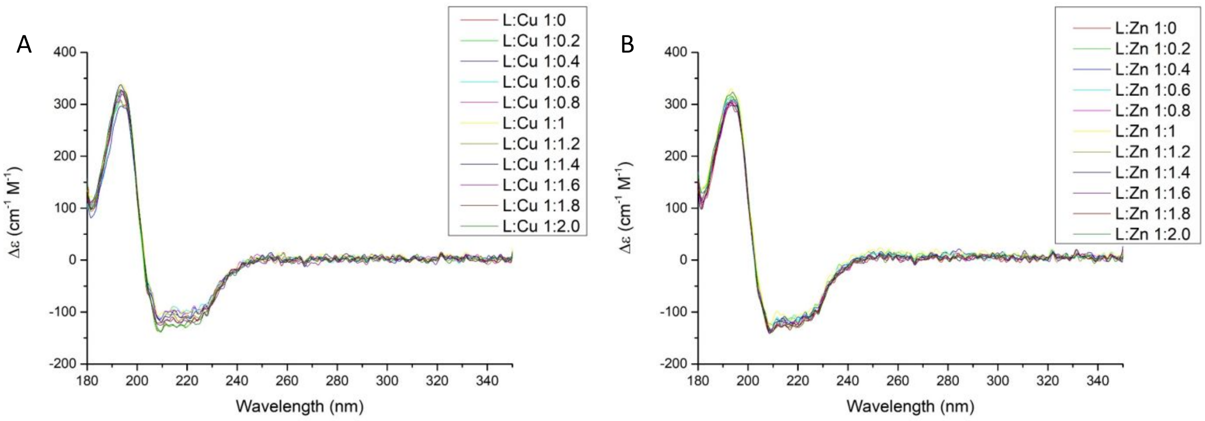

Fig. S10: Secondary structure of piscidin 1 and its copper(II) (A) and zinc(II) (B) complexes. In both cases, the addition of metal ions does not cause any significant changes.

Table S1. Examples of MIC breakpoints values from EUCAST Version 14.0 [1] for bacteria.

Enterobacterales (for *E. coli*)	MIC breakpoints (µg/mL)		*Enterococcus* spp. (for *E. faecalis*)	MIC breakpoints (µg/mL)		*Staphylococcus* spp. (for *S. aureus*)	MIC breakpoints (µg/mL)	
	S≤	R>		S≤	R>		S≤	R>
Amoxicillin-clavulanic acid iv.	8	8	Ampicillin	4	8	Amikacin	8	8
Piperacillin	8	8	Ampicillin-sulbactam	4	8	Chloramphenicol	8	8
Piperacillin-tazobactam	8	8	Amoxicillin	4	8	Fosfomycin *iv*	32	32
Ticarcillin	8	16	Amoxicillin-clavulanic acid	4	8	Nitrofurantoin	64	64
Ticarcillin-clavulanic acid	8	16	Nitrofurantoin	64	64			
Cefadroxil	16	16						
Cefalexin	16	16						
Fosfomycin *iv*	32	32						
Fosfomycin oral	32	32						
Nitrofurantoin	64	64						
Nitroxoline	16	16						

REFERENCES

[1] The European Committee on Antimicrobial Susceptibility Testing, Breakpoint tables for interpretation of MICs and zone diameters, 14th ed., 2024. https://www.eucast.org/clinical_breakpoints/.
